# FAT1 functions as an oncogenic driver in triple negative breast cancer through AKT pathway-driven effects on the matrisome

**DOI:** 10.7150/ijbs.104921

**Published:** 2025-02-24

**Authors:** Panpan Zhao, Yuanyuan Zhang, Yang Yu, Qing Zhang, Xiaoying Liu, Xu Dong Zhang, Song Chen, Charles E. de Bock, Rick F. Thorne, Yujie Shi

**Affiliations:** 1 Translational Research Institute, Henan Provincial People's Hospital, Zhengzhou University, Zhengzhou 450003, China.; 2 Department of Breast Surgery, Henan Provincial People's Hospital, People's Hospital of Zhengzhou University, People's Hospital of Henan University, Zhengzhou, Henan Province, 450003, China.; 3 Children's Cancer Institute, Lowy Cancer Research Centre, UNSW Sydney, Sydney, NSW, Australia.; 4 School of Clinical Medicine, UNSW Sydney, Sydney, NSW, Australia.; 5 Department of Pathology, People's Hospital of Zhengzhou University, Zhengzhou 450003, Henan, China.; 6 Academy of Medical Sciences, Zhengzhou University, Zhengzhou 450000, Henan, China.

**Keywords:** triple negative breast cancer, FAT1, matrisome, PI3K-AKT signaling, integrin switching

## Abstract

FAT1 cadherin exhibits dual tumor suppressor and oncogenic roles across various cancers, but its function in breast cancer remains unclear due to conflicting reports of mutational loss and overexpression. In this study, we demonstrate that FAT1 mRNA and protein levels are reduced during mammary transformation, an effect linked to promoter methylation rather than mutational events. Subtype-specific analysis reveals that high FAT1 expression correlates with poor outcomes in basal-like/triple-negative breast cancer (TNBC), while elevated FAT1 expression in luminal A/estrogen receptor-positive breast cancers is associated with improved patient prognosis. Functional studies in TNBC models using knockdown and overexpression approaches confirm that FAT1 promotes both cell proliferation and motility. High-throughput sequencing and biochemical assessments establish strong links between FAT1 phenotypes and the activation of PI3K-AKT signaling. Additionally, FAT1 manipulation induces significant changes in matrisome-related genes, extracellular matrix components, and integrin switching. Together, these findings define an oncogenic role for FAT1 in TNBC, providing mechanistic insights into how its regulation influences AKT signaling, cell proliferation, and motility.

## Introduction

Breast cancer accounts for ~12% of global malignancies and now rivals lung cancer as the most commonly diagnosed cancer type 1, 2. Cases are typically divided into three pathological categories: estrogen receptor (ER) and progesterone receptors (PR)-positive cases, human epidermal growth factor 2 (HER2)-positive (overexpressing) cases, and lastly triple negative breast cancers (TNBC), so named because of deficiency of ER and/or PR expression, along with lack of HER2 overexpression 3. Notably, TNBC accounts for ~20 percent of all breast cancer diagnoses and is considered the most aggressive subtype with high rates of early recurrence and distant metastases 4-6. In particular, the extensive intratumoral heterogeneity of TNBC complicates its treatment where the lack of specific targeting agents leads to unsatisfactory outcomes 7, 8. Therefore, exploring key molecular targets and signaling pathways in TNBC can potentially provide new diagnostic markers and targets towards improving patient outcomes.

This study focuses on FAT1, one of four Fat genes belonging to the cadherin superfamily 9, 10. First identified in Drosophila, *Fat* was ascribed tumor suppressor functions 11 with the first mammalian homolog *FAT1* being cloned from human leukemia cells 12, 13. Early reports showed FAT1 functioned to regulate cell polarity, adhesion, and migration with interactions detailed between the Fat1 cytoplasmic tail and intracellular scaffold proteins 14-16. For example, Fat1 influences actin dynamics during cell adhesion and motility, binding to Ena/VASP proteins 17 and affecting their recruitment to focal adhesions 15, 16. Notably, these hallmark effects on adhesion and migration suggest that Fat1 is implicitly involved in regulating cellular interactions with the extracellular matrix (ECM), although to date no definitive extracellular Fat ligands in vertebrates have been identified. Other reports made links with cell proliferation control. For example in zebrafish, Scribble binding to the Fat1 cytoplasmic tail inhibits Yap1, the central regulator of the Hippo pathway, thereby affecting cell growth 18. Cytosolic FAT1 interactions with β-catenin also serve to limit its nuclear translocation, affecting Wnt signal transduction with ensuing effects on cell proliferation 14, while a subsequent report highlighting a link between the presence of FAT1 mutations in cancer and aberrant Wnt pathway activation 19. The latter study arguably brought to prominence the relationship between FAT1 mutation and tumor biology.

Numerous allelic studies reported deletions or loss of heterozygosity of the FAT1 locus in a variety of cancer types 9. Later sequencing evidence revealed somatic mutations associated with FAT1 inactivation, particularly in squamous cell carcinomas arising at different body sites 20-22. Indeed, FAT1 was shown to function as a disease driver in this context, serving as a platform to assembles a Hippo kinase 'signalome' complex that otherwise moderates downstream YAP1 signaling 22. Moreover, genetic loss of *Fat1* predisposes mice to chemically-induced skin squamous carcinogenesis with further insights provided into the consequences of YAP1 activation accompanying FAT1 loss wherein cells enter a hybrid-EMT state, promoting tumor stemness and other key malignant characteristics 23. In ER-positive breast cancers FAT1 deletion similarly rewires the Hippo pathway, promoting elevated CDK6 levels and resistance to CDK4/6 inhibitors 24. Related work in TNBC cells showed that reducing FAT1 expression through proteasomal degradation serves to activate the Hippo pathway and promote cancer cell stemness and chemotherapy resistance 25. Nonetheless, these findings must be balanced against other reports showing overexpression of FAT1 mRNA and protein in breast cancer 13, 26, akin to gliomas and carcinomas of the cervix, colorectum, liver, pancreas and blood system where FAT1 has been proposed to exert a pro-cancer role 9, 10.

With these reports in mind, we revisited the topic of FAT1 in breast cancer to examine clinicopathological associations. We observed diversification among breast cancer subtypes where FAT1 was most highly expressed in TNBC/Basal-like breast cancers where its higher expression was associated with worsened patient outcomes. Moreover, high FAT1 expression was found to align with treatment response signatures with bioinformatic analyses suggesting that FAT1 is involved in establishing an immunosuppressive microenvironment within breast cancer lesions. Empirical assessment in TNBC cell lines revealed that FAT1 imparts primary oncogenic functions, increasing cellular proliferative and invasive activity in association with of the PI3K/AKT signalling pathway activation. These activities show a high degree of concordance with modifications to the “matrisome”, a gene classification system broadly encompassing proteins making up the extracellular matrix (ECM), along with related cellular receptors such as integrins which act as intermediary links between the cytoskeleton and the ECM 27, 28. Manipulating FAT1 resulted in both compositional changes in structural and regulatory ECM elements along with altered integrin expression profiles, reflecting integrin switching. Together these findings provide new perspectives regarding the role of FAT1 in breast cancer, also laying a foundation for further exploration of the role of FAT1 in the development and progression of TNBC.

## Materials and Methods

### Bioinformatic analyses

Comparisons of FAT1 mRNA/protein expression and promoter methylation in normal mammary and breast cancer tissues together with pathological and molecular classifications, and other clinical associations were undertaken using the indicated publicly available data and tools. Website links and key analysis parameters are provided in the text and figures.

### Breast cancer cell lines and culture

Human breast cancer cell lines (MDA-MB-231, MDA-MB-468, Hs578t, MCF7) were cultured in Dulbecco's Modified Eagle Medium (DMEM) (C3103-0500; BI) supplemented with 10% fetal bovine serum (FBS; BI). Alternatively, BT549 cells were maintained in Roswell Park Memorial Institute (RPMI)-1640 medium (C3001-0500; BI). The human mammary epithelial cell line MCF10A was cultured in Mammary Epithelial Cell Medium (CM-0525, Procell, China). All cell lines were sourced as previously described 29 and their identities confirmed using STR profiling. Routine mycoplasma tests were conducted to ensure the cell lines were negative (D101-01, Vazyme, China).

### Inducible shRNA knockdown and overexpression of FAT1 by transfection

Inducible knockdown was accomplished using lentiviral-mediated transduction of control or FAT1 targeting shRNAs using the FH1-tUTG vector in combination with the pMDLg.pRRE, pMD2.g and pRSU.pREV packaging plasmids as previously described 30. Briefly, lentiviral particles were prepared by transfecting HEK293T cells (Lipofectamine 2000, 11668030, Invitrogen) for 48h and subjecting the conditioned medium to filtration through 0.45 μm non-pyrogenic filters. Fresh supernatants were immediately incubated with target cells culture in 6 well plates in the presence of 8 μg/ml Polybrene (TR-1003-G, Sigma-Aldrich). Virus containing media was removed after 48 h and replaced with fresh growth medium containing puromycin. Functional experiments were performed in the presence of 2 µg/ml DOX (25316-40-9, Sigma-Aldrich) to initiate FAT1 knockdown. For overexpression, full length codon optimized FAT1 cDNA cloned into the pcDNA3.1 plasmid (synthesized by GeneArt) was transfected into TNBC cells using Lipofectamine 2000 according to the manufacturer's recommendations (Tables S1 and S2).

### Quantitative polymerase chain reaction (PCR) assays

Total RNA was extracted using the SPARKeasy Tissue/Cell RNA kit (AC0201, Sparkjade) and reverse transcribed into cDNA using the HiScript III RT SuperMix RT reagent Kit (R323, Vazyme) according to the manufacturer's instructions. Quantitative real-time PCR (qRT-PCR) was performed on a Step One™ real-time fluorescent quantitative PCR system (Thermo Fisher Scientific) using 2×SYBR Green qPCR Mix (AH0101, Spark jade). Actin served as the reference gene for data normalization using the ΔΔCt method. Primer sequences are provided in Table S3.

### Western blotting

Cleared cell lysates were prepared using RIPA buffer supplemented with protease (P001; NCM Biotech) and phosphatase inhibitors, respectively (P003; NCM Biotech). Protein concentrations were measured using a BCA Protein Assay kit (PC0020; Solarbio) and equal protein amounts separated by electrophoresis using 4-12% gradient or 10% SDS-PAGE gels (Future PAGE, ACE) before transfer onto nitrocellulose (NC) membranes (0.22 μm, Millipore). Membranes were then blocked with skim milk in TBST buffer for 1 h at RT, and subsequently incubated with primary antibodies overnight at 4 °C and then secondary antibodies for 1h at RT. Bands were detected using enhanced chemiluminescence system (ED0015-C, Sparkjade) with images collected using a ChemiDoc Imager (BioRad). Antibodies are listed in Table S4.

### Cell proliferation assays

Cell growth/viability assays were conducted using the Cell-counting Kit 8 (CCK-8; C0005, TargetMol). Briefly, cells were plated in a 96-well culture plates at 1000 cells per well in standard 10% FBS culture medium before adding the CCK-8 reagent the indicated times and absorbance at 450 nm measured using a microplate reader (Thermo Scientific™ Varioskan™ LUX). Alternatively, cell clonogenicity was evaluated in colony formation assays where 1000 cells were seeded into 6-well plates and cultured for 10-14 days, replenishing with fresh culture media every 4 days. After rinsing the wells twice in ddH2O, the colonies were fixed in methanol for 10 min, rinsed again in ddH2O before staining with 0.4% crystal violet. After drying, the wells were imaged and Image J software used to estimate total colony counts and areas.

### Tumor sphere assay

MDA-MB-231 cells were seeded at 1000 cells/well into ultra-low adhesion 6 plates (3471; Corning) in serum-free Complete MammoCult™ Medium (STEMCELL Technologies) and cultured for 12 days. Total tumor spheres >100 μm in diameter were recorded in each well along with sphere diameters. Three replicate wells were included in each experiment for each cell group.

### Cell motility assays

Scratch (wound-healing) assays were performed on cells cultured to reach ~95% confluency. Wound tracks were created by scraping the cell monolayer with 200μl pipette tips and gently removing the detached cells by washing three times with PBS. The cells were subsequently cultured in serum-free culture medium and wounds sequentially imaged over 0-48 h with changes in the empty area measured using Image J software. Alternatively, Transwell migration assays were conducted using 8μm pore cell culture inserts (3422; Corning). Cells (2.5×10^4^) in 200μL medium without FBS were first seeded into the upper chambers while 600 µL medium containing 20% FBS was added to the lower chamber. After cultured for 24h, the media was removed and cell culture inserts were fixed in 4% formaldehyde for 30 min followed by staining with 0.4% crystal violet for 30 minutes. At least 5 different fields of cells migrating to the bottom membrane were imaged/well and cell counts performed using Image J software.

### Transcriptome sequencing and analysis

Total RNA was extracted using Trizol reagent (15596018, Thermo Fisher) following the manufacturer's procedure with the RNA quantity and purity analyzed with a Bioanalyzer 2100 instrument using the RNA 6000 Nano LabChip Kit (5067-1511, Agilent). High-quality RNA samples with RIN number > 7.0 were used to construct sequencing libraries. Briefly, mRNA was purified from total RNA (5ug) using Dynabeads Oligo (dT) (Thermo Fisher) with two rounds of purification. Following purification, mRNAs were fragmented for 5-7min at 94℃ using divalent cations (Magnesium RNA Fragmentation Module, e6150, NEB). The cleaved RNA fragments were then reverse-transcribed to cDNA using SuperScript™ II Reverse Transcriptase (1896649, Invitrogen) before synthesizing U-labeled second-stranded DNAs with a mixture of *E. coli* DNA polymerase I (m0209, NEB), RNase H (m0297, NEB) and dUTP Solution (R0133, Thermo Fisher). An A-base was then added to the blunt ends of each strand, preparing them for ligation to the indexed adapters. Each adapter contained a T-base overhang for ligating the adapter to the A-tailed fragmented DNA. Dual-index adapters were ligated to the fragments, and size selection was performed with AMPureXP beads. After the heat-labile UDG enzyme (m0280, NEB) treatment of the U-labeled second-stranded DNAs, the ligated products were amplified with PCR by the following conditions: initial denaturation at 95℃ for 3 min; 8 cycles of denaturation at 98℃ for 15 sec, annealing at 60℃ for 15 sec, and extension at 72℃ for 30 sec; and then final extension at 72℃ for 5 min. The average insert size for the final cDNA libraries were 300±50 bp. Lastly, 2×150bp paired-end sequencing (PE150) was conducted using Illumina Novaseq™ 6000 (LC-Bio Technology CO., Ltd., Hangzhou, China) following the vendor's protocol. After sequencing, the data was filtered to obtain high-quality (clean) data for comparison against the human reference genome. Gene differential expression analysis was performed by DESeq2 software between two different groups (and by edgeR between two samples). Genes with the parameter of false discovery rate (FDR) below 0.05 and absolute fold change ≥ 2 were defined as differentially expressed genes (DEGs). DEGs were then subjected to enrichment analysis for KEGG and GSEA pathways along with comparisons against the Matrisome gene set derived from 28. Bioinformatic analyses were performed using the OmicStudio tools at https://www.omicstudio.cn/tool. Diagrams were drawn based on the R (https://www.r-project.org/) on the OmicStudio platform (https://www.omicstudio.cn/tool).

### Confocal microscopy

Cells grown on glass coverslips were fixed in 4% formaldehyde for 30 min at RT and thereafter blocked with 5% BSA-PBS solution for 1 h at RT before incubation with anti-vinculin antibodies diluted at 1:1000 overnight at 4 °C. Cells were washed in PBS, incubated with Alexa Fluor 594 (Red) conjugated secondary antibodies at a dilution of 1:1000 (A11005; Invitrogen) for 1h. Cell cytoskeleton (filamenous actin) was stained with Oregon Green® 488 phalloidin (Green) at a dilution of 1:1000 (O7466; Invitrogen), and cell nuclei decorated with 4',6-diamidino-2-phenylindole (DAPI). Confocal images were collected with a HC PlanApo 63X (NA 1.4) oil objective using a Leica DMi8 fitted with the SP8 confocal scanning system.

### Reproducibility and statistical analyses

Experiments were performed independently at least three times. Differences between experimental groups were assessed using GraphPad Prism with statistical tests comparing two groups involving the two-tailed Student's t test while ANOVA tests were used for multiple group/parameter comparisons. P values less than or equal to 0.05 were considered to represent statistically significant differences.

## Results

### Mutational status of FAT1 in breast cancer

To explore the paradigm of *FAT1* loss in breast cancer, we began by analyzing its cell-type- expression in the breast. Immunohistochemical staining of normal breast tissues showed low to moderate signals for FAT1 protein evident in epithelium including luminal epithelial (secretory) and myoepithelial (basal) cells along with endothelial staining 31 (Fig. S1A-D). Consistently, data mining of publicly available single cell analysis data 32 verified FAT1 prominent FAT1 mRNA expression occurred in each major breast epithelial cell type (Fig. S1E). Thus, FAT1 is clearly detectable in secretory epithelial cells whose subsets are considered the cell of origin of breast cancer 33.

**Figure 1:**
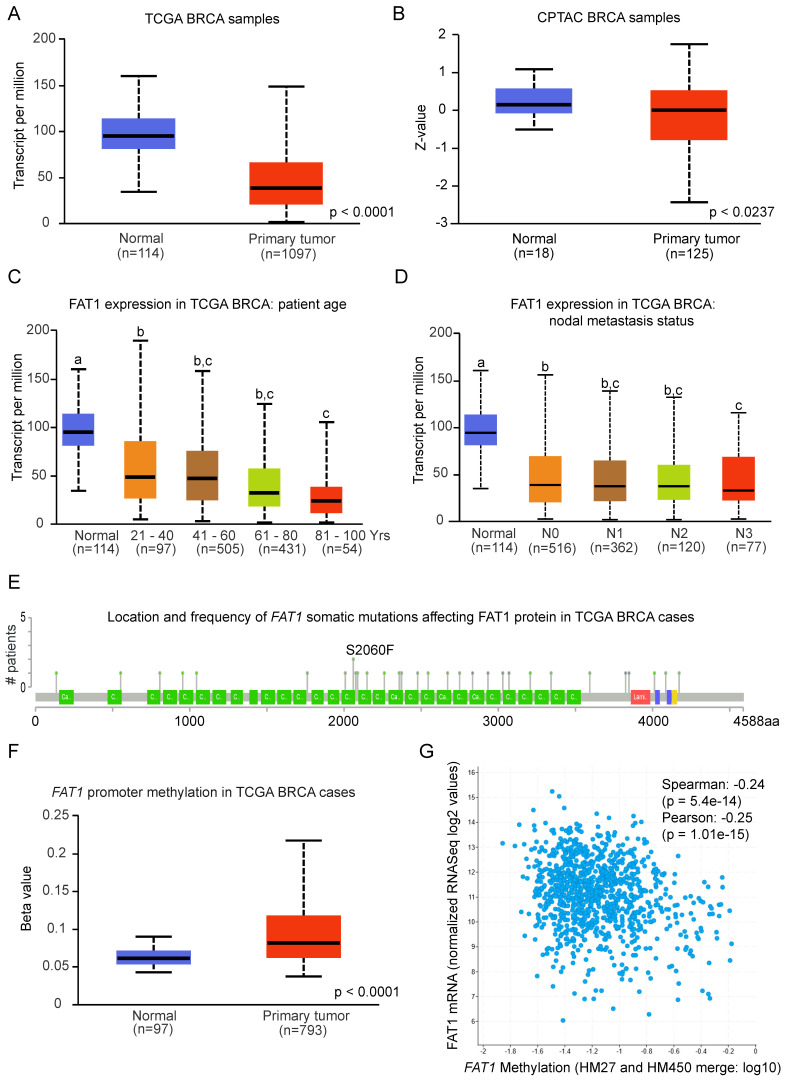
** FAT1 expression and mutation status in breast cancer tissues. (A, B)** Box and Whisker plots comparing FAT1 mRNA (A) and protein (B) expression levels from normal breast and primary breast cancer tissue samples deposited in the TCGA and CPTAC databases, respectively. Z-values represent standard deviations from the median across samples using normalized log2 spectral count ratio values. Data were obtained from interrogating the UALCAN platform (ualcan.path.uab.edu) 76. Statistical differences determined using Student's t-test. **(C, D)** Depiction of FAT1 mRNA expression in tissues analyzed as per (A) according to patient age of diagnosis (C) and nodal metastasis status (N0, no metastasis; N1, N2 and N3, metastasis to 1-3, 4-9 and 10 or more axillary nodes, respectively). Means are not statistically significant for columns marked with the same letter. **(E)** Location of individual mutations within the FAT1 protein sequence in samples from the TCGA Breast Invasive Carcinoma dataset (G) plotted using cBioportal (recurring S2060F mutations are highlighted). **(F, G)** FAT1 promoter methylation levels in normal breast and primary breast cancer tissue samples from the TCGA dataset analysed using the UALCAN platform (F). Statistical differences determined using Student's t-test. Correlation between FAT1 mRNA expression and FAT1 gene methylation levels in 977 samples in the TCGA Breast Invasive Carcinoma dataset plotted using cBioportal (www.cbioportal.org) 77) (G).

We then considered whether FAT1 was differentially expressed in normal versus primary breast cancer tissues. Cross comparisons of the breast cancer datasets from the TCGA and GTEx resources showed decreased FAT1 mRNA expression in malignant tissues (Fig. 1A). This expression pattern was also reflected in FAT1 protein level differences between normal and tumor tissues (Fig. 1B). Notably, expression in primary breast tumors was not related to nodal metastasis status, although higher expression was biased towards younger patients (Fig. 1C, D), the latter of interest given that age biases exist in pathological/molecular subset classifications (refer below).

To investigate the mechanisms driving differential FAT1 expression following transformation, we examined its mutational status and promoter methylation levels. Only ~5% of breast cancer cases had *FAT1* mutations, with gene amplifications and deep deletions being rare while missense mutations (either shallow or diploid) were more common including a small number of truncating mutations (Fig. S1F). Notably, except for two samples sharing an S2060F mutation, all somatic mutations were unique (Fig. 1E). Taken together, the low frequency and lack of recurrent *FAT1* mutations is unlikely to be responsible for the changes in FAT1 mRNA expression between normal and breast cancer tissues. Rather, analysis of promoter methylation in *FAT1* showed increases in breast tumor versus normal comparisons and consistently, FAT1 mRNA expression was negatively correlated with promoter methylation levels (Fig. 1F, G). However, the methylation beta values for tumor tissues fall below those considered to delineate a hyper-methylated state. In sum, these findings indicate *FAT1* expression decreases with breast cancer transformation and is associated with increased promoter methylation.

### Differential expression of FAT1 in breast cancer subsets

**Figure 2:**
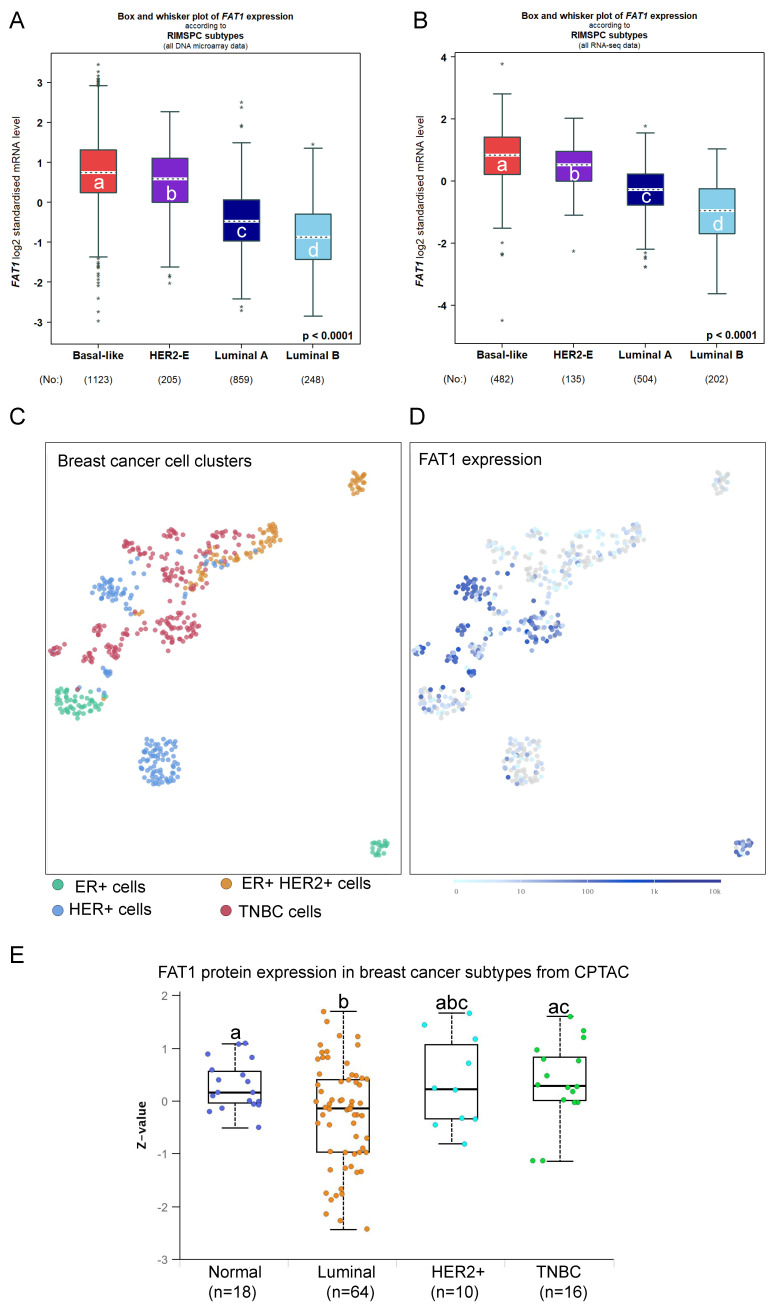
** FAT1 expression in breast cancer subtypes. (A, B)** FAT1 mRNA expression in breast cancer molecular subtypes classified by RIMSPC (robust intrinsic molecular subtype predictors classification) definitions from DNA microarray (A) and RNA-seq (B) based studies. Means are not statistically significant for columns marked with the same letter. Plots prepared using the Breast Cancer Gene-Expression Miner v5.0 (bc-GenExMiner v5.0: http://bcgenex.ico.unicancer.fr/) 40-42. **(C, D)** UMAP plot showing cell type clusters defining breast cancer histological subtypes cells isolated from primary breast cancer cells and lymph node metastases (C; 520 cells from n=11 patients) with the corresponding plot of FAT1 expression (ENSG00000083857; D). Data derived from Nguyen *et al.* 78 interrogated using the EMBL-EBI Single Cell Expression Atlas: ebi.ac.uk). **(E)** Comparative FAT1 protein levels among major breast cancer subclasses and normal breast tissues. Means are not statistically significant for columns marked with the same letter. Plots were derived from CPTAC data interrogated using UALCAN (ualcan.path.uab.edu 76).

The preceding findings prompted us to examine associations between FAT1 expression and breast cancer pathological and molecular classifications. Analyses based on microarray and RNA-seq technologies, respectively, revealed that ER positive breast tumors expressed the lowest FAT1 levels while those cases lacking ER showed higher FAT1 expression (Fig. S2A, B). Smaller differences occurred among histological classifications although IDC cases recorded the highest overall FAT1 expression levels (Fig. S2C, D). Regarding intrinsic molecular classifications defined by gene profiling 34, the highest FAT1 expression was associated with Basal-like and Normal-like cases along with HER2+ with lowest expression occurring in the Luminal B subtype using Hu's classification 35 (Fig. S2E, F). Similar findings were evident using the Sorlie and PAM50 classifiers (data not shown). Other simplified schemes such as RIMSPC (robust intrinsic molecular subtype predictors classification; 36, 37), SCMGENE, SCMOD1 and SCMOD2 also showed that the highest and lowest FAT1 expression was associated with the basal-like and luminal B subsets, respectively (Fig. 2A, B, and data not shown). In general agreement with these findings, SCA analysis of primary breast cancer tissues showed that FAT1 expression predominately aligned with cancer cells from TNBC and HER2 cases but not the luminal A and luminal B subtypes (Fig. 2C, D). Furthermore, it can be noted there was a high degree of concordance between data from the microarray and RNA-seq studies, lending confidence to these findings. Nevertheless, not all genes display good correlation between mRNA and protein levels in breast cancer tissues 38, but importantly we found the subtype variations in FAT1 mRNA expression were maintained at the protein level with the highest levels in TNBC (Fig. 2E).

TNBC are encompassed within the ER/PR negative classification and furthermore there is a substantial overlap of TNBC and basal-like breast cancers 34, 39. The inherent heterogeneity of TNBC cases has prompted different subclassification schemes 34 including those developed by Burstein *et al.* 39. These signatures consist of four subtypes, LAR (luminal androgen receptor), mesenchymal (MES), basal-like immune-activated (BLIA) and basal-like immunosuppressed (BLIS) subtypes. Stratification of TNBC cases according to FAT1 among these subtypes showed that highest expression occurred in the BLIS grouping (Fig. S2G, H). Thus, based on pathological and molecular features, high FAT1 expression in breast cancer is most closely associated with ER/PR negative and TNBC/basal-like cases while lowest FAT1 expression occurs the ER+ and luminal B classifications.

### Prognostic significance of FAT1 in breast cancer

We next sought to determine if FAT1 expression differences impacted patient outcomes. Towards addressing this question, we accessed microarray and RNA-seq data deposited in the Breast Cancer Gene-Expression Miner v5.0 (bc-GenExMiner v5.0; 40-42 and derived Kaplan Meier plots.

**Figure 3:**
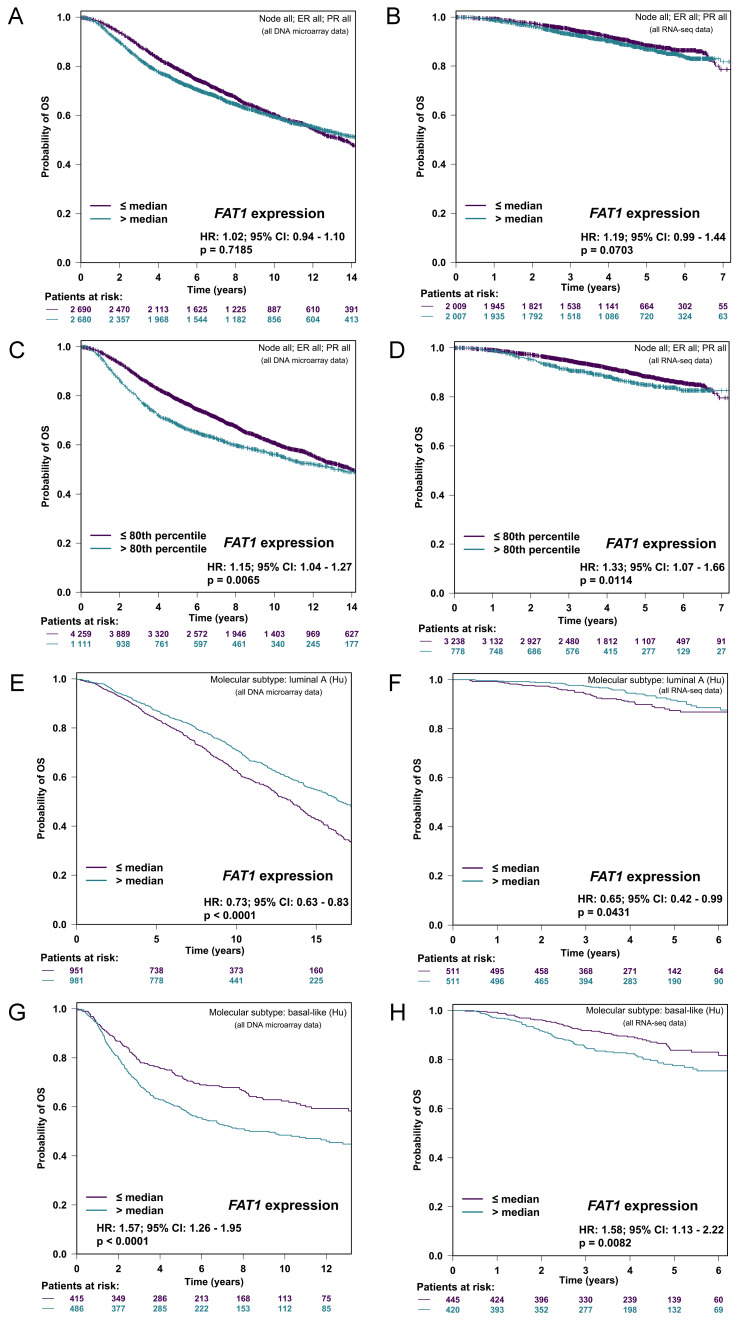
** FAT1 expression and breast cancer patient prognosis. (A-D)** Kaplan-Meier plots of overall survival (OS) in breast cancer patients stratified according to FAT1 mRNA expression in DNA microarray (A, C) and RNA-seq (B, D) based studies. Plots prepared with bc-GenExMiner v5.0 40 using median (A, B) and optimal (C, D) cutoff settings. P values obtained using the log-rank test. **(E-H)** Kaplan-Meier plots of OS in patients stratified according to median FAT1 mRNA expression in the Luminal A (E, F) and Basal-like molecular subtypes (G, H) classified using Hu's definitions. Plots prepared with bc-GenExMiner v5.0 from DNA microarray (E, G) and RNA-seq (F, H) based studies. P values obtained using the log-rank test.

First, using a conservative median cut-off approach to stratify patients according to mRNA levels, FAT1 expression was not associated with overall survival (OS) in either microarray or RNA-seq-based patient cohorts (Fig. 3A, B). Nonetheless, repeating the analyses with an optimal cutoff indicated significantly worse OS for the 20% of cases with the highest FAT1 expression (Fig. 3C, D). Subdividing cases according to Hu's molecular subtype classifications indicated high FAT1 expression was associated with a survival advantage in the Luminal A classification whereas high FAT1 expression predicated worse OS in Basal-like tumors (Fig. 3E-F). No differences were detected in either the Luminal B or HER2 classifications (Fig. S3A, B) with mostly similar findings made employing other classification schemes (Fig. S3C, D). On balance, these findings suggest that different patient outcomes are associated with FAT1 expression according to breast cancer subtype.

**Figure 4:**
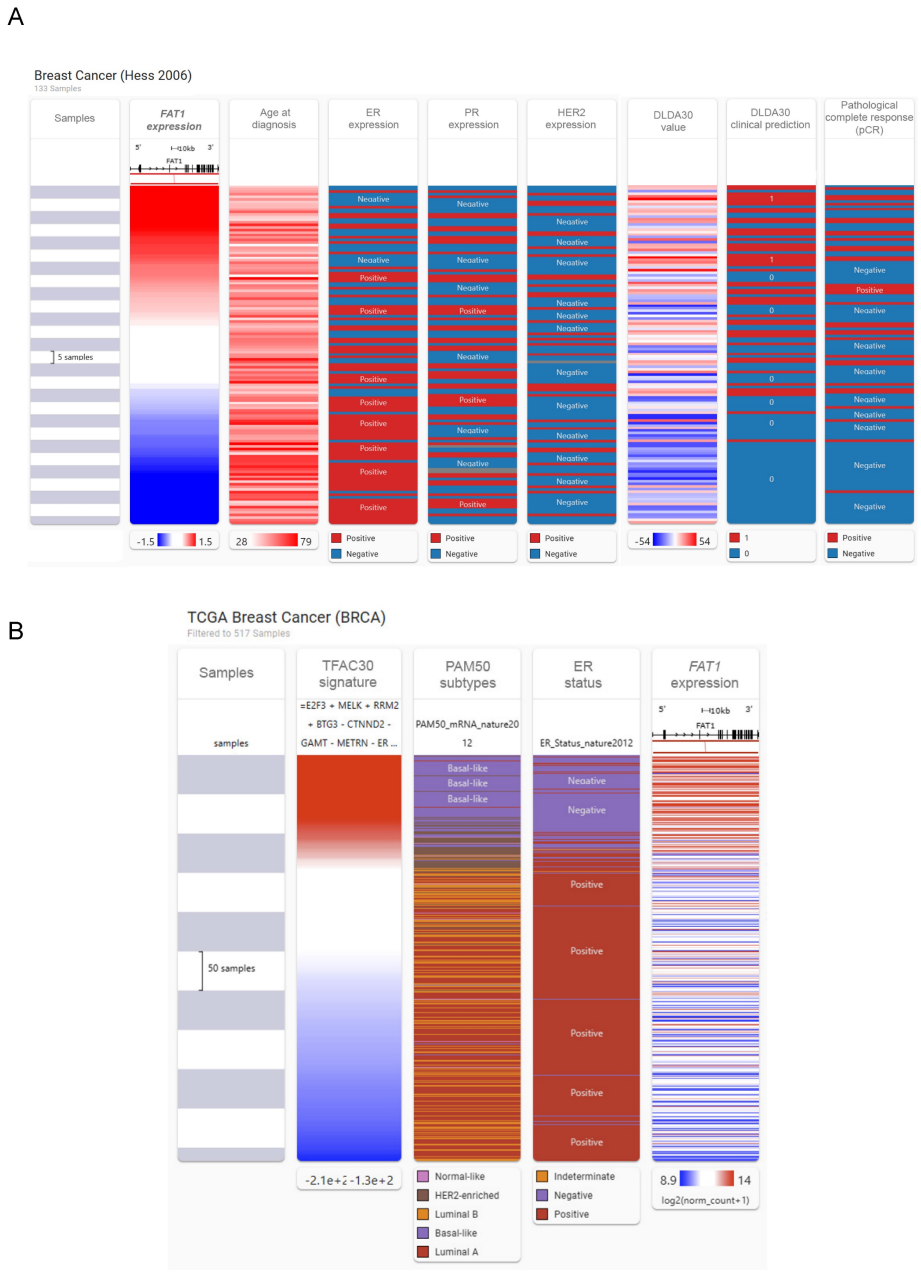
** Association between FAT1 expression and responses to chemotherapy. (A)** Comparative heatmaps ranking breast cancer cases according to FAT1 expression against age of diagnosis, ER, PR and HER2 expression, the DLD30A chemotherapy response signature values, clinical prediction scores and response outcomes to first line chemotherapy. Data were generated from the Hess *et al.* microarray study 44 using the USCA Xena platform (xenabrowser.net) 43. **(B)** Comparative heatmaps derived from the TCGA BRCA dataset as per (A) ranking breast cancer cases according to the TFAC30 chemotherapy response signature values against PAM50 subtype attributions, ER expression status and FAT1 mRNA expression.

We then investigated associations between FAT1 and other clinicoprognostic features. As described above, resistance to CDK4/6 inhibitors accompanies FAT1 loss in ER+ cases 24, raising a broader question of relationships between FAT1 and therapeutic responses. We considered this point using a multiomic approach comparing FAT1 expression and clinical phenotype data in breast cancer using the USCA Xena platform 43. Comparative heatmaps were derived from the Hess *et al.* study by ranking cases using FAT1 expression from microarrays. Notably, this study developed a 30 gene classifier (DLDA30 a.k.a. TFAC30) that predicts responses to preoperative chemotherapy using paclitaxel, fluorouracil, doxorubicin and cyclophosphamide chemotherapy (T/FAC) 44. This analysis showed general features consistent with our preceding findings where high FAT1 expressing tumors tended to be from young patients and lack ER expression with mostly an ambivalent distribution among PR and HER2 positive cases (Fig. 4A). Moreover, there were obvious overlaps between high FAT1 expression and TFAC30 values predicting treatment outcomes as well as positive responses recorded for chemotherapy. Applying the predictive signature to RNAseq-based TCGA data showed similar strong alignments between positive TFAC30 scores and high FAT1 expressing BRCA tumors along with demonstrating clear alignment with Basal-like tumors based on PAM50 and ER negative status (Fig. 4B).

We also considered if FAT1 was associated with tumor-immune system interactions within in breast tumors using the TISIDB web portal 45. Among the different subtypes in the TCGA database, FAT1 gave strong scoring associations with immune subtypes in breast cancer (BRCA) (Fig. S4A) with its high expression most closely associated with the TGF-β dominant immune subclassification (Fig. S4B). Indeed, BRCA along with ovarian cancer (OV) showed the most positive correlations between FAT1 and range of immunomodulatory molecules including TGF-β and TGF-βR1 (Fig. S4C, D). Moreover, compared with other cancers, FAT1 expression in BRCA showed the strongest positive enrichment with CX3CL1 and numerous CXCL cytokines (Fig. S4E, F). Notably, the latter collective of genes influences inflammation and immune responses in tumors, many of which are frequently linked with cancer progression through various mechanisms affecting tumor and non-tumor cells in the tumor microenvironment (TME) 46. Similarly, BRCA was among the top cancer types for positive enrichment of tumor-infiltrating lymphocytes (TILs) with a strong score for T-helper 2 (Th2) cells (Fig. S4G, H) which have been equated with establishing an immunosuppressive TME in breast and other cancers 47.

Collectively these findings propose that FAT1 influences outcomes in patients in a subtype specific manner with high FAT1 expression in the TNBC correlating with the worsened outcomes. Nevertheless, whether high FAT1 is a coincidental marker of TNBC or otherwise contributes to its tumorgenicity required clarification.

### FAT1 promotes the proliferative phenotype of TNBC cells

Assessment of a large panel of breast cancer cell lines using data from the Neve study 48 using the GOBO platform 49 indicated similar patterns of expression to *ex vivo* tissues with TNBC cases showing the highest FAT1 mRNA levels, followed by HER2 positive lines while ER/PR+ lines recorded the lowest expression (Fig. S5A). Western blotting corroborated these findings with some TNBC cell lines demonstrating high levels of FAT1 protein (BT549, MDA-MB-231 and Hs578t) compared to other TNBC cells (MDA-MB-468 and HCC38), ER+ cells (MCF-7) and normal mammary cells (MCF10A) (Fig. S5B). On this basis, we selected the BT549, MDA-MB-231 and Hs578t lines to conduct further analyses assessing the contribution of FAT1 to TNBC tumorigenicity.

Employing lentiviral-mediated transduction with a doxycycline-inducible shRNA gene knockdown system, we screened five independent shRNA hairpins designed to target FAT1 (sh#1-sh#5) mRNA, comparing their effects relative to a non-targeting control (sh#C). This analysis identified sh#2 and sh#5 as being relatively more effective than other shRNAs at reducing FAT1 mRNA and protein levels in different TNBC cell lines (Fig. S5C, D) and we subsequently used these for further functional experiments.

**Figure 5:**
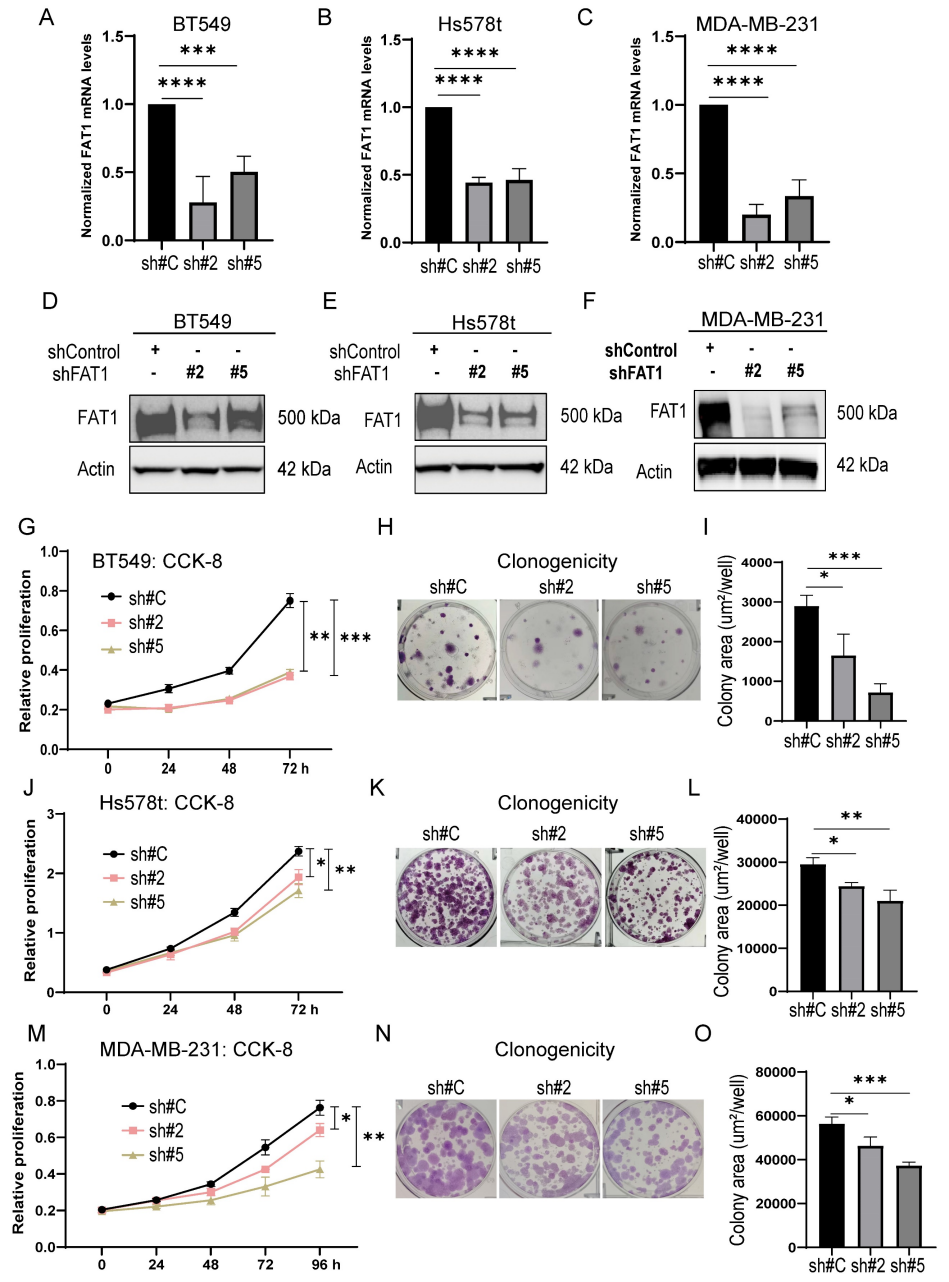
** FAT1 knockdown inhibits TNBC cell growth. (A-F)** FAT1 mRNA (A-C) and protein (D-F) levels measured by qPCR and Western blot, respectively, in BT549 (A, E) and Hs578t (B, E) and MDA-MB-231 (C, F) cell lines transduced with doxycycline (DOX) inducible shRNAs targeting FAT1 (sh#2, sh#5) or a control shRNA (sh#C). Cells were treated with DOX for the duration of the experiments. **(G-O)** The proliferative capacity of cells from (A-F) measured in CCK-8 assays (G, J, M) and colony formation assays. Representative colony images (H, K, N) and quantitation of colony growth (I, L, O). Reproducibility and statistical tests. (A-O) Results representative of three independent experiments. (A-C, G, I, J, L, M, O) Data shown as mean ± SD of three replicates with statistical differences analyzed by one way ANOVA (A-C, I, L, O) or two way ANOVA (G, J, M). *P < 0.05; **P < 0.01; ***P < 0.001; ****P < 0.0001.

Measuring the impact of FAT1 expression on the proliferative capacity of TNBC cells using CCK-8 and clonogenicity assays showed that knockdown of FAT1 in BT549, Hs578t and MDA-MB-231 cells (Fig. 5A-F) resulted in significant diminishment of growth and clone forming ability, respectively (Fig. 5G-O). Importantly, there was good consistency between the results of independent targeting shRNAs, indicative that the effects likely resulted from specific targeting. Second, we implemented the reverse approach to increase FAT1 levels in TNBC cells by transfection. Opposite to knockdown effects, ectopic FAT1 expression in BT549 cells promoted increased proliferation and colony formation (Fig. S5E-I). Collectively, these findings indicate that FAT1 expression positively affects TNBC cell proliferation.

Lastly, given FAT1 deletion has been reported to promote cancer stem cells (CSCs) in squamous carcinomas 23, we further looked at whether altered FAT1 levels in TNBC also affects tumor stemness. Towards this, we assessed tumor spheroid formation which is considered the gold standard *in vitro* assay for testing the self-renewal ability of cancer stem cells 50. MDA-MB-231 cells bearing either the control shRNA or FAT1 shRNAs were cultured at low density in stem cell media before measuring the number and size of tumor spheres after 12 days. We found that the tumor spheroids following FAT1 knockdown were not significantly diminished in number although they presented as significantly smaller than the control shRNA cultures (Fig. S5J-L). Western blotting analysis against stemness markers showed that KLF4, c-MYC, OCT4A, NANOG, and SOX2 were not overtly affected by FAT1 knockdown in MDA-MB-231 cells although most markers were decreased in equivalent assays of BT-549 cells (Fig. S5M). Thus, effects on proliferation rather than stemness are responsible for the deficient growth of MDA-MB-231 cells following FAT1 knockdown. Furthermore, while cell type specific effects on stemness marker expression were evident in BT-549 cells, these changes indicate reduced rather than increased stemness associated with FAT1 depletion.

### FAT1 promotes TNBC cell migration and invasion

**Figure 6:**
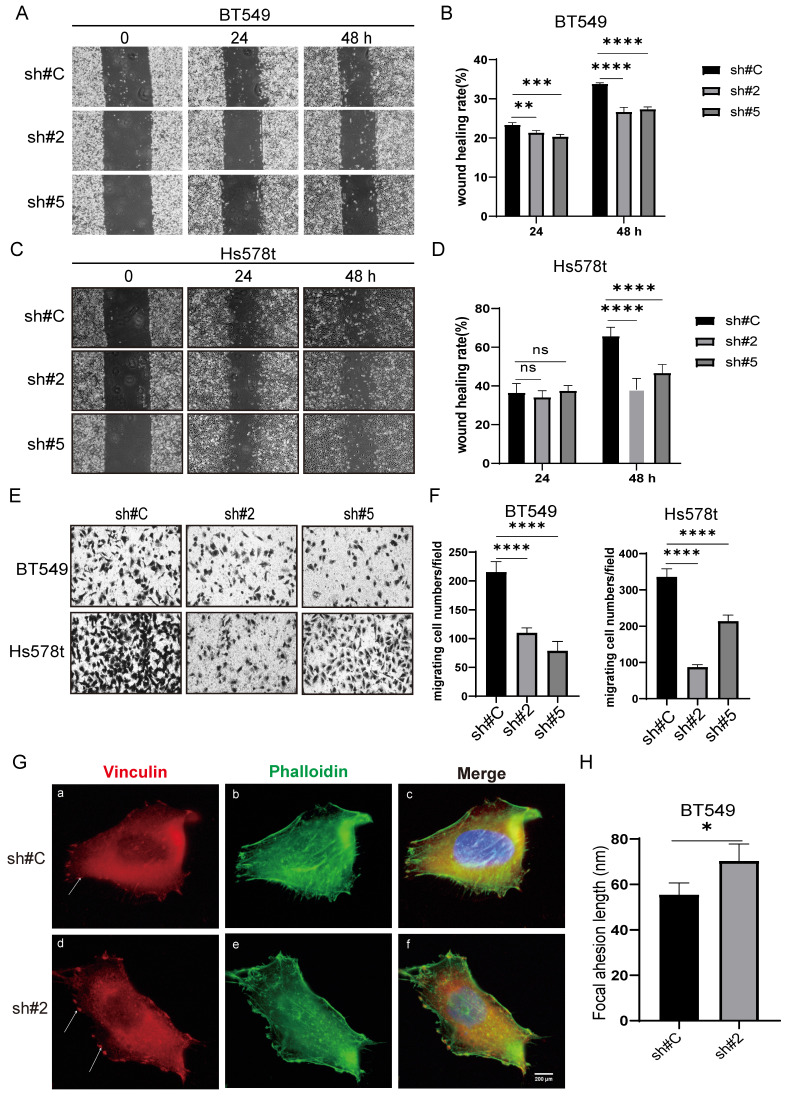
** FAT1 knockdown inhibits the migratory and invasive capacity of TNBC cells. (A-D)** Wound healing assays measuring cell motility were conducted on the BT549 (A, B) and Hs578t (C, D) cell lines transduced with doxycycline (DOX) inducible shRNAs targeting FAT1 (sh#2, sh#5) or a control shRNA (sh#C). Representative images collected of the same area at 0, 24 and 48h (A, C) and quantitation of cell migration distance as wound healing rate (B, D). **(E, F)** Transwell migration assays measuring cell invasion were conducted on the BT549 and Hs578t cell lines from (A-D). Migrating cells were fixed and stained after 24h and images collected (E) and quantitated as the number of cells per microscopic field (F). **(G, H)** BT549 cells transduced with shRNAs targeting FAT1 (sh#2) or a control shRNA (sh#C) were stained with antibodies against vinculin (red) and phalloidin (green) to decorate focal adhesions and filamentous actin, respectively. After counterstaining cell nuclei with DAPI (blue), images were collected using confocal microscopy. Representative images of cells (G) with quantitation of focal adhesion length (H). Reproducibility and statistical tests. (A-H) Results representative of three independent experiments. (B, D, F, H) Data shown as mean ± SD of three replicates with statistical differences analyzed by one way ANOVA (B, D, F) or Student's t test (H). *P < 0.05; **P < 0.01; ***P < 0.001; ****P < 0.0001.

Using the same manipulations as above, we extended our analysis to assess the contribution of FAT1 to TNBC tumor cell metastatic potential using wound healing and Transwell invasion assays. Similar findings were derived from BT549 and Hs578t cell lines where FAT1 silencing resulted in significant decreases in cell motility in wound healing capabilities along with reduced invasion in Transwell assays (Fig. 6A-F). Repeating the analysis following ectopic FAT1 expression in different TNBC cell lines resulted in relative increases in these assays (Fig. S6A-H). Together these results indicate that FAT1 expression promotes cell motility, which in a tumor context may reflect effects on cancer metastasis. To ascertain if this mechanism was related to the altered migratory phenotype observed in TNBC cells, we examined whether there were FAT1-dependent changes in the cortical actin network and focal adhesion structures. Indeed, we observed that the filamentous actin network revealed by phalloidin staining was less organized following FAT1 knockdown. Moreover, this accompanied the appearance of larger (longer) vinculin-decorated focal adhesion plaques (Fig. 6G, H), the latter structures being critical for cell adhesion and migration upon the extracellular matrix (ECM). Such phenotypic changes following FAT1 knockdown are wholly consistent with the cell phenotype of altered (reduced) motility.

### Transcriptomic analysis uncovers links between FAT1 and PI3K/AKT1 signaling

**Figure 7:**
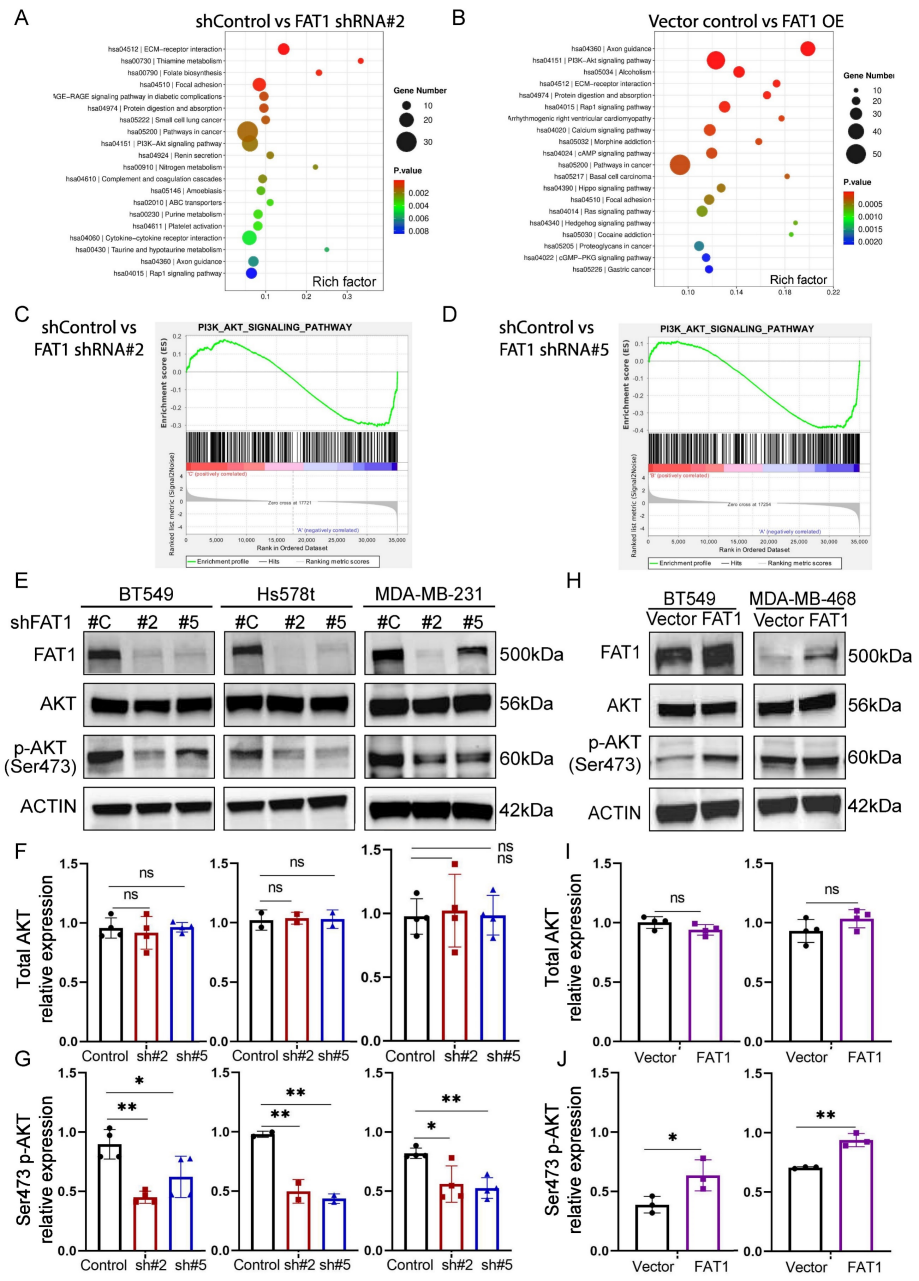
** Transcriptomic analyses of TNBC links FAT1 manipulation with PI3K-Akt signalling. (A, B)** KEGG enrichment bubble plots of the top ranked pathways altered following the knockdown (A; shFAT1#2) or overexpression (OE) of FAT1 (B) in BT459 cells. **(C, D)** GSEA enrichment plots for the PI3K_AKT-SIGNALING_PATHWAY gene signature changes comparing independent comparisons of control shRNA versus FAT1 knockdown (shFAT1#2, C; shFAT1#5, D). **(E-J)** Western blotting analyses comparing changes in FAT1, total and Ser471 phosphorylated AKT and conducted in the indicated TNBC cell lines following knockdown using shRNA (E) or overexpression of FAT1 (H). Relative changes in total and Ser473 phosphorylated AKT were determined by densitometric quantitation of bands relative to the actin loading control from the knockdown (F, G) and overexpression (I, J) experiments, respectively. Results representative of three independent experiments (E, G) Data shown as mean ± SD of three replicates with statistical differences analyzed by one way ANOVA (F, G) or Student's t test (I, J). *P < 0.05; **P < 0.01; ***P < 0.001; ****P < 0.0001.

Next, to glean clues regarding the underlying mechanisms involving FAT1 phenotypes in TNBC, we undertook whole transcriptome sequencing of FAT1 knockdown and overexpression BT549 cells, respectively. This analysis identified 796 differentially expressed genes following FAT1 knockdown, of which 282 genes were upregulated and 514 were downregulated, respectively (Tables S5 and S6). In comparison, 1600 differentially expressed genes were detected after FAT1 overexpression, including 1074 upregulated and 526 downregulated genes, respectively (Table S7). We then implemented KEGG and GSEA analyses to characterize gene changes influenced by FAT1. Among the top KEGG pathway enrichments associated with both FAT1 knockdown and overexpression, the strongest common enrichment in terms of total gene numbers was the 'Pathways in Cancer' signature followed by the 'PI3K-Akt signaling pathway' (Fig. 7A, B). High probability scores were also enriched for more focused signatures including the 'ECM-receptor interaction pathway' together with the inclusion of several signatures related to cell ECM interactions and cell motility processes including 'Axon guidance', 'Focal adhesion', and the 'Rap1 signaling pathway', all of which are considered crucial for regulating cell polarity, migration and adhesion as well as proliferation. Other notable inclusions occurring in one of the two pathway lists were the 'Hippo' and 'Hedgehog' signaling pathways, the former being notable from previous literature.

Visualization of the GSEA pathway enrichment profiles for the PI3K-AKT pathway indicated significant downregulation following FAT1 knockdown (Fig. 7C, D). Consistently, using Ser473 phosphorylated AKT as a proxy for PI3K/mTORC2/AKT activation, we found that FAT1 knockdown in three different TNBC cell lines was accompanied by the downregulation of Akt signaling (Fig. 7E-G and Fig. S7C). Furthermore, overexpression of FAT1 resulted in Akt pathway activation (Fig. 7H-J and Fig. S7D), proposing a general positive relationship between the levels of FAT1 and Akt signaling state.

**Figure 8:**
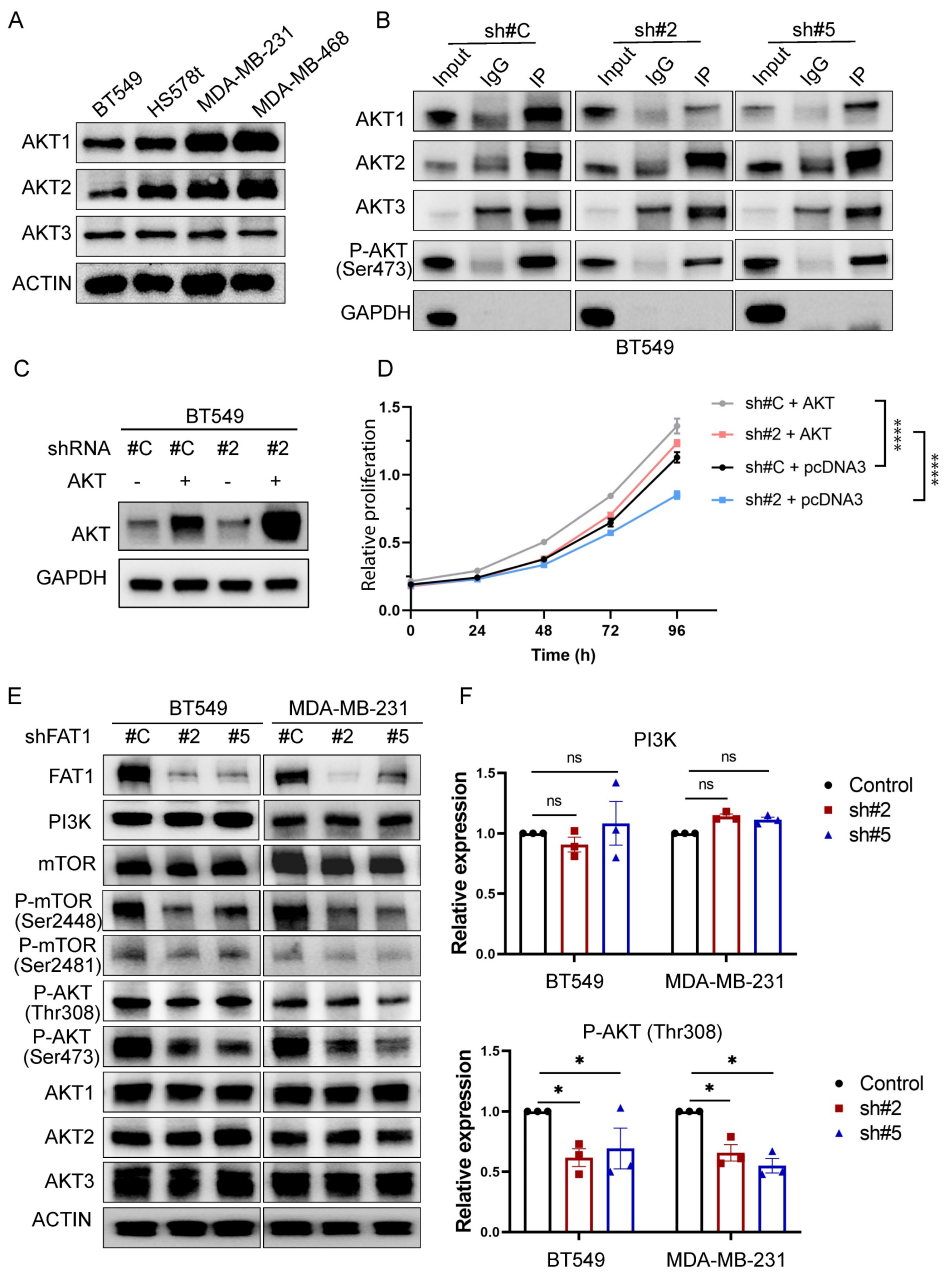
** Dual effects of FAT1 on mTORC1 and mTORC2 activation in TNBC cells. (A)** Western blotting analyses comparing the expression of AKT isoforms in the indicated TNBC cell lines. Actin was used throughout as a loading control. **(B)** BT549 cells transduced with control (sh#C) or FAT1 targeting shRNAs (sh#2) were subject to immunoprecipitation using control IgG or anti-p-Ser473 AKT antibodies before immunoblotting against AKT isoforms (AKT1, AKT2, AKT3) and a GAPDH negative control. **(C, D)** BT549 cells transduced with control (sh#C) or FAT1 targeting shRNAs (sh#2) were transfected with a control (pcDNA3) or AKT1 expression vector. Western blotting against total AKT confirmed AKT overexpression (C) with the proliferative capacity of cells from (C) measured in CCK-8 assays. Data shown as mean ± SD of three replicates with statistical differences determined by two-way ANOVA (***P < 0.001 for control versus test conditions as indicated). **(E, F)** Western blotting analyses comparing the expression of FAT1, PI3Kα, total and phosphorylated (Ser 2448 and Ser 2481) mTOR, total and phosphorylated (Ser 473 and Ser 308) AKT and AKT isoforms in control versus FAT1 shRNA knockdown BT549 and MDA-MB-231 cells (E). Relative changes in PI3K and p-Ser308 AKT levels determined by densitometric quantitation of bands relative to the actin loading control (F). Data shown as mean ± SD of three experiments with statistical differences analyzed by one way ANOVA (ns, not significant, *P < 0.05).

Since phospho-Ser473 antibodies have pan-specificity for AKT1, AKT2, and AKT3, it was relevant to identify which isoform(s) were influenced by FAT1. Consistent with previous reports 51, we found the TNBC cell lines used in this study all co-express AKT1, AKT2, and AKT3 (Fig. 8A). To delineate isoform-specificity, we analyzed the Ser473-phosphorylated pool of AKT recovered by immunoprecipitation. Notably, AKT1 was the main isoform showing diminished Ser473 phosphorylation following FAT1 knockdown in BT549 cells (Fig. 8B). Additionally, we found that overexpression of AKT1 in BT-549 knockdown cells served to rescue growth inhibition in FAT1 shRNA knockdown cells (Fig. 8C, D). Thus, these findings suggest that AKT1 is principally involved in mediating FAT1-dependent growth signals in TNBC cells. We further observed reductions in Ser308 AKT phosphorylation accompanying FAT1 knockdown with reductions in both mTOR Ser2481 and Ser2448 phosphorylation (Fig. 8E, F), the latter reflecting reduced mTORC1 activity. No overall effects were observed on total PI3Kα, mTOR or AKT isoform levels in these experiments. Together, these findings suggest that FAT1 contributes to the oncogenic phenotype of TNBC cells via a PI3K/AKT1/mTORC2 axis, with mTORC1 activation also playing an interconnected role in the FAT1 signaling network.

### Links between FAT1, PI3K/Akt signaling and matrisome regulation

Delving into the changes associated with the PI3K-AKT signaling pathway enrichment showed numerous downstream gene targets encoding extracellular matrix proteins including collagens (COL1A1, COL4A1, COL4A2, COL4A3, COL4A4, COL6A3, COL9A1, COL9A2), laminins (LAMA1, LAMB1, LAMC2, LAMA2, LAMA3), thrombospondins (THBS2, THBS3) and fibronectin (FN1) along with related secreted factors and cell adhesion molecules (namely integrin genes ITGA2, ITGA4, ITGA7, ITGA10, ITGB3, ITGB4, ITGB5, ITGB6, ITGB8, ITGB11) (Fig. S7A, B). Given this striking observation, together with the associations between FAT1 and ECM-receptor interactions and related pathways, we then characterized the FAT1 cell phenotypes based on the "matrisome" definition (Table S8).

**Figure 9:**
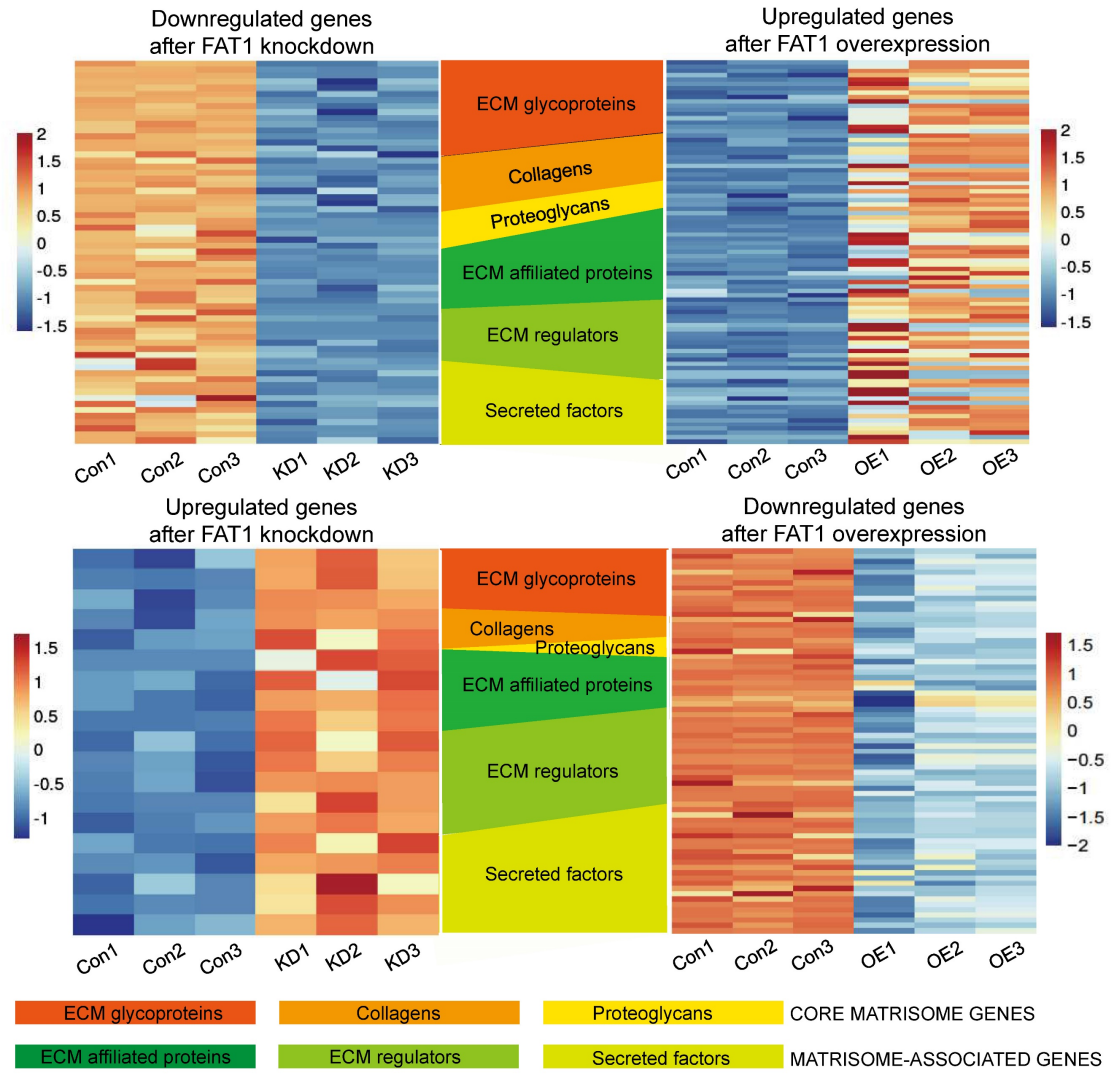
** Changes in matrisome-related genes in TNBC following manipulation of FAT1.** Heatmaps showing patterns of change in core matrisome (ECM glycoproteins, Collagens, Proteoglycans) and matrisome-related (ECM affiliated proteins, ECM regulators, Secreted factors) genes in BT549 cells. Comparisons between downregulated genes after FAT1 knockdown (shCtrl versus FAT1 sh#2) and upregulated genes after FAT1 overexpression (vector versus FAT1) (top). Comparisons between upregulated genes after FAT1 knockdown (shCtrl versus FAT1 sh#2) and downregulated genes after FAT1 overexpression (vector versus FAT1) (bottom).

**Figure 10:**
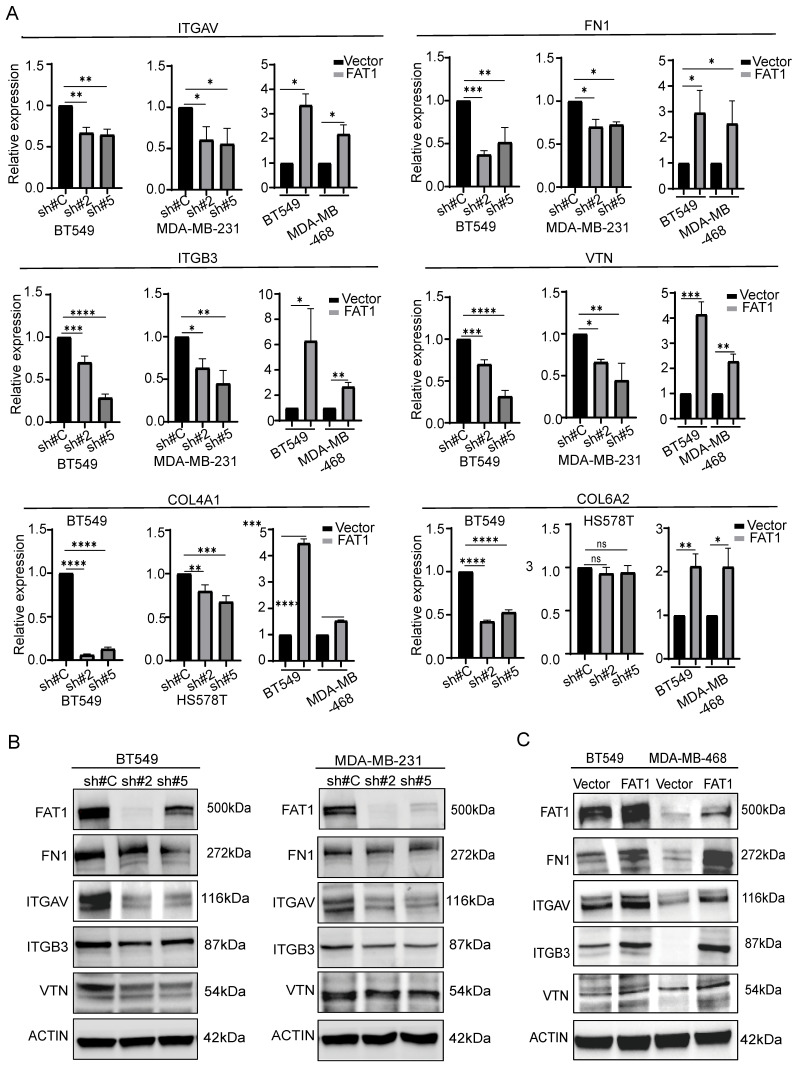
** Changes in matrisome-related genes in TNBC following manipulation of FAT1. (A)** Comparative changes in matrisome-related gene transcripts (ITGAV, FN1, ITGB3, VTN, COL4A1, and COL6A2) measured by qPCR in the indicated TNBC cell lines following FAT1 knockdown or overexpression. **(B, C)** Comparative changes in matrisome-related proteins (FN1, ITGAV, ITGB3, and VTN) measured by Western blot in the indicated TNBC cell lines following FAT1 knockdown (B) or overexpression (C). Reproducibility. (A-C) Results representative of three or more independent experiments.

The matrisome includes two major subcategories: core matrisome components involving ECM glycoproteins, collagens and proteoglycans along with matrisome-associated genes encompassing other ECM affiliated proteins which include cell adhesion molecules such as integrins, extracellular matrix regulatory factors and other secretory factors. Accordingly, we analyzed our differentially expressed gene sets according to these categories. Visualizing the results with the OmicStudio (https: //www.omicstudio.cn/tool) bioinformatics analysis tool indicated there were numerous intersections between genes altered by FAT1 expression and genes belonging to each of the matrisome subcategories (Fig. 9). We subsequently implemented qPCR assays against selected cell adhesion genes (αv and β3 integrin subunits encoded by *ITGAV* and *ITGB3*, respectively) along with the ECM components fibronectin (FN1), vitronectin (VTN), and collagens (COL4A1 and COL6A2). This analysis showed that αv and β3 integrin subunit expressions were diminished upon FAT1 depletion while ectopic FAT1 increased their expression. And except for COL6A6, which was not changed upon FAT1 knockdown in Hs578t cells, mRNA levels of FN1, VTN, COL4A1 and COL6A2 all followed the same pattern of changes (Fig. 10A). Western blotting analyses for ITGAV, ITGB3, FN1 and VTN largely showed their protein level changes largely phenocopied the effects of FAT1 manipulation on their mRNA levels (Fig. 10B, C and Fig. S8A, B).

## Discussion

Early Drosophila studies described enlarged “*fat*” flies with later work linking overgrowth phenotypes with disrupting gene mutations. Other evidence identified *fat* as a negative regulator of the Hippo pathway 52, cementing *a priori* that vertebrate Fat cadherins function as tumor suppressor genes. However, this notion is oversimplified as functional differences exist between the two Drosophila *fat* homologues. Moreover, the imperfect fidelity in protein domain organization between Drosophila and mammalian Fat genes 53 inherently suggests functional diversification. Indeed, despite Fat4 being considered the true *fat* ortholog, Fat1 has been closely aligned with Hippo pathway signaling in vertebrates. However, the literature regarding FAT4 is generally limited compared to FAT1, particularly cancer related studies.

Of relevance to breast cancer, it is notable that Fat4 deletion was reported to transform murine mammary cells 54. Moreover, breast cancer was one of the more obvious cancer types showing reduced FAT4 expression among TCGA datasets 55 with reduced FAT4 expression in TNBC tissues along with *in vitro* findings consistent with tumor suppressor function 56. With respect to FAT1, as noted in the Introduction, some cancers such as pancreatic adenocarcinomas overexpress FAT1 57 while studies in breast cancer provide mixed conclusions, with reports of mutational loss contrasting with findings of overexpression. This study now serves to reconcile these differences wherein subtype-specific effects of FAT1 accommodate both oncogene and tumor suppressor functions.

FAT1 is clearly detectable in secretory epithelial cells whose subsets are considered the cell of origin of breast cancer 33 while its overall levels appear reduced in breast cancer tissues. Nonetheless, the broad range of FAT1 tumor expression compared to normal tissues presumably reflects the spectrum of FAT1 loss and overexpression. Further analyses showed significant variations in FAT1 expression according to pathological classifications with highest expression in ER-/PR- cases with lower expression in ER+ cases. Even stronger differences were evident using molecular classification schemes where FAT1 expression trended highest in Basal-like/TNBC tumors, followed by HER2+, Luminal A and Luminal B cases. Stratifying patients according to FAT1 expression uncovered divergent effects on patient overall survival with worsened outcomes for TNBC cases with high FAT1 expression while the opposite was found for Luminal A. As discussed below, FAT1 likely fulfils the duties of a traditional oncogene in TNBC, endorsing key functions of tumor progression. Other intriguing associations were made regarding chemotherapy response signatures where high FAT1 expression was associated with responses to first-line chemotherapy, a characteristic feature of the clinical course of TNBC where good initial treatment responsiveness is followed by acquired resistance and aggressive relapse 58.

Dissection of TNBC subtypes showed that highest FAT1 expression occurs in the BLIS subtype, a classification associated with the worst survival outcomes (Burstein, *et al.* 2015). Notably, the BLIS signature encompasses downregulated immune response genes along with cell cycle progression and DNA repair components. Other analyses provided concordance with the impact of FAT1 on the immune landscape of breast cancer where among different cancer types, FAT1 expression recorded notable associations with key immunomodulatory molecules such as TGF-β and TGF-βR1, together with a range of cytokines including CX3CL1 and other CXCL members, as well as influencing key types of tumor-infiltrating immune cells. Collectively these findings predicate a highly immunosuppressive tumor microenvironment (TME): TGF-β signaling is known to promote progression in established lesions through inhibitory effects on immune cells 59; the cytokines enriched with FAT1 also influence inflammation and immune responses in tumors 46; and the increase in infiltrating lymphocytes such as Th2 cells has been equated with immunosuppression in breast and other cancers 47. It is relevant to note our analyses are based on bulk cell populations and the source of FAT1 expression may not only be tumor cells as stromal cell types including endothelial cells and fibroblasts express FAT1 53. On the other hand, it is improbable that FAT1 signals represent immune cells as mature hematological lineages express low or negligible FAT1 levels 12, 60. Links between FAT1 and immunosuppression have been documented in glioma where high FAT1 expression drives TGF-β1 production 59 while another study in non-small cell lung cancer reached similar conclusions 61. Moreover, immunotherapy appeared more effective in colorectal cancer patients whose tumors express mutated rather than wildtype FAT1, a phenomenon strongly associated with the PI3K-AKT signature 62. Thus, high FAT1 expression in TNBC cells plausibly facilitates immunosuppression although this concept needs to be formally demonstrated in appropriate models.

We found that FAT1 maintains the proliferative phenotype of TNBC cells as well as promoting their invasive capacity, contrasting with the reported tumor suppressive actions of FAT1 in some cancers 9, 10 as well as FAT4 in TNBC 56. Nevertheless, the links between FAT1 and TNBC cell motility are fully consistent with early reports describing Fat1's role in controlling actin dynamics via engagement with Ena/VASP proteins 15, 16. Our knockdown and overexpression experiments produced corroborative findings with transcriptomic analyses disclosing a common link to the Pathways in Cancer and PI3K-AKT Signaling signatures. The former encompasses a spectrum of receptor-driven cellular processes which converge on proliferative and migratory circuits, ranging from cytokine-cytokine receptor interactions, ECM-receptor interactions and focal adhesion, chemokine signaling through GPCRs, Wnt and TGFβ signaling, Notch and Hedgehog pathways, the death receptor pathway, together with BCR and Toll-like receptor signaling. Notably, the Pathways in Cancer signature also incorporates PI3K-AKT signaling whose regulatory state correlated with the cell phenotypes observed, and whose broad contributions in conferring malignant characteristics has prioritized it as a target in breast and other cancer types 63.

PI3K-AKT signaling is classically linked with the epithelial-mesenchymal transition (EMT) and although TNBC cells express EMT markers, more recent thinking to accommodate cancer cell plasticity has led to the hybrid EMT concept. Namely that cancer cells co-express both epithelial and mesenchymal markers, providing advantageous characteristics such as stemness and drug resistance 64. A recent report showed that reductions in FAT1 promote TNBC cell stemness in association with the induction of Sox2 25. In contrast, we observed that the diminished proliferation accompanying FAT1 knockdown in BT-549 was associated with decreased stemness marker levels including Sox2, although curiously not affecting the stem potential of MDA-MB-231 cells. Notably, the latter cells are directly comparable to the Bu *et al.* study 25, although the methods differ since we did not pre-enrich for stem-like cells. In any event, we found that FAT1 expression produced an associated oncogenic phenotype among the different TNBC cell lines analyzed. Here, we linked the growth phenotype to isoform specific effects of AKT1 activation through the PI3K/mTORC2 axis. While all three AKT isoforms have overlapping roles in growth control, arguably the strongest links are between AKT1 and breast cancer proliferation 65, with AKT3 also contributing significantly in TNBC 51. Furthermore, we also found that the FAT1 signaling network impacted PI3K/mTORC1 activation, possibly resulting from effects on feedback loops that exist within the mTOR network or changes in upstream regulators of AKT, although how this precisely occurs will require further investigation.

Notably, many changes denoting the FAT1-AKT pathway association in TNBC conspicuously involved matrisome-related genes, dovetailing with the striking impact of FAT1 on various cell adhesion and motility associated pathways. Guided by this observation, we illustrated how FAT1 expression influences TNBC cells from a matrisome perspective. This treatment expands the view of FAT1 beyond regulation of actin dynamics and polarity regulation, identifying broad changes in core matrisome genes including many structural components such as collagens, fibronectin and vitronectin. In turn, these form ligands for adhesion receptors including integrins, enabling cells to engage with the extracellular matrix, often in the context of focal adhesion complexes. The latter are dynamic structures supporting cell adhesion and movement, also acting as signaling platforms 66, 67. Notably, our findings are compatible with early reports linking Fat1 with the control of actin dynamics via the engagement of Ena/VASP, which affects focal adhesions 15, 16. Moreover, this aligns with the role of PI3K-AKT signaling, for example, invoking PI3K activity in breast cancer cells serves to activate FAK (focal adhesion kinase), increasing the levels of αvβ3 integrin and promoting cisplatin resistance 68. Furthermore, the PI3K-AKT-dependent actions on motility may represent a generalized pathway in cancers with FAT1 overexpression, for example, as reported in high-grade gliomas whose invasiveness was dependent upon EGFR-AKT signaling 69.

Our results further impinge upon the concept of 'integrin switching', a phenomenon involving changes in the expression of integrin αβ pairs/pairings which provides cells with altered abilities to engage with the ECM 70. Interestingly, integrin switching has been linked with EMT in TNBC where gains in β3 integrin expression serve to alter TGF-β signaling and reinforce the EMT program 71. Moreover, CDH3 (cadherin 3) present in the cell-cell junctions of 'leader' cells were recently shown to organize the collective migration of breast cancer through effects on integrins as well as the localized secretion of laminin 72. Other research suggests that high αvβ3 expression in TNBC defines stem-like properties towards ensuring genomic stability and resistance to PARP inhibition 73. Both observations draw interesting parallels to findings here where αv and β3 integrin levels were positively modulated through FAT1. Notably, changes to integrin expression and ECM structural components were not the only significant alterations encountered. For instance, important ECM modifiers such as matrix metalloproteases (MMPs and ADAMs), serpins and complement factors are also featured. Similarly, expression changes in interleukins, CXCL cytokines, TGFβ3, and BMPs—key components of TGFβ signaling—along with WNT ligands, highlight important mechanisms linking high FAT1 expression to poor outcomes in TNBC. These findings together with FAT1's role in promoting immunosuppressive effects provide substance for further investigations.

As a corollary to our findings, a relevant question concerns cause and effect relationships between FAT1 TNBC phenotypes and the processes identified by our analysis. Despite its heritage, FAT1 has yet to be proven to function in cell adhesion, at least in the manner of classical cadherins. Neither is it a transcription factor *per se*, although its cleavage products participate in the regulation of mitochondrial function 74, 75, as well acting in the nucleus as transcriptional co-factors 14. Whether or not the changes in TNBC cell proliferation are linked to such metabolic regulation, or if the transcriptional changes are attributable to FAT1 fragments remains an open question. In any case, while most cell adhesion molecules do not directly encode signaling domains, they are involved in signaling as exemplified through bidirectional signaling processes involving integrins whose engagement of ECM protein ligands is not only essential for cytoskeletal network connections but also to engage various signaling pathways. For instance, integrins regulate pathways including Ras and Rho-GTPase, Wnt, Notch, and Hippo to exert effects on cell motility, invasion, and migration 66. Indeed, FAT1-induced changes to all such pathways are evident from our transcriptomic data, and while a common denominator involved AKT activation, multifaceted reasons are most likely to underly the changes in TNBC phenotypes. In any case, our findings offer new opportunities for unravelling the complex contributions of FAT1 towards TNBC biology.

## Supplementary Material

Supplementary figures.

Supplementary tables.

## References

[1] Siegel RL, Miller KD, Wagle NS, Jemal A (2023). Cancer statistics, 2023. CA Cancer J Clin.

[2] Bray F, Laversanne M, Sung H, Ferlay J, Siegel RL, Soerjomataram I (2024). Global cancer statistics 2022: GLOBOCAN estimates of incidence and mortality worldwide for 36 cancers in 185 countries. CA Cancer J Clin.

[3] Zhu Z, Jiang L, Ding X (2023). Advancing Breast Cancer Heterogeneity Analysis: Insights from Genomics, Transcriptomics and Proteomics at Bulk and Single-Cell Levels. Cancers (Basel).

[4] Lee J (2023). Current Treatment Landscape for Early Triple-Negative Breast Cancer (TNBC). J Clin Med.

[5] Liedtke C, Mazouni C, Hess KR, André F, Tordai A, Mejia JA (2023). Response to Neoadjuvant Therapy and Long-Term Survival in Patients With Triple-Negative Breast Cancer. J Clin Oncol.

[6] Lehmann BD, Bauer JA, Chen X, Sanders ME, Chakravarthy AB, Shyr Y (2011). Identification of human triple-negative breast cancer subtypes and preclinical models for selection of targeted therapies. J Clin Invest.

[7] Vagia E, Mahalingam D, Cristofanilli M (2020). The Landscape of Targeted Therapies in TNBC. Cancers (Basel).

[8] So JY, Ohm J, Lipkowitz S, Yang L (2022). Triple negative breast cancer (TNBC): Non-genetic tumor heterogeneity and immune microenvironment: Emerging treatment options. Pharmacol Ther.

[9] Sadeqzadeh E, de Bock CE, Thorne RF (2014). Sleeping giants: emerging roles for the fat cadherins in health and disease. Med Res Rev.

[10] Chen ZG, Saba NF, Teng Y (2022). The diverse functions of FAT1 in cancer progression: good, bad, or ugly?. J Exp Clin Cancer Res.

[11] Mahoney PA, Weber U, Onofrechuk P, Biessmann H, Bryant PJ, Goodman CS (1991). The fat tumor suppressor gene in Drosophila encodes a novel member of the cadherin gene superfamily. Cell.

[12] de Bock CE, Ardjmand A, Molloy TJ, Bone SM, Johnstone D, Campbell DM (2012). The Fat1 cadherin is overexpressed and an independent prognostic factor for survival in paired diagnosis-relapse samples of precursor B-cell acute lymphoblastic leukemia. Leukemia.

[13] Dunne J, Hanby AM, Poulsom R, Jones TA, Sheer D, Chin WG (1995). Molecular cloning and tissue expression of FAT, the human homologue of the Drosophila fat gene that is located on chromosome 4q34-q35 and encodes a putative adhesion molecule. Genomics.

[14] Hou R, Liu L, Anees S, Hiroyasu S, Sibinga NE (2006). The Fat1 cadherin integrates vascular smooth muscle cell growth and migration signals. J Cell Biol.

[15] Moeller MJ, Soofi A, Braun GS, Li X, Watzl C, Kriz W (2004). Protocadherin FAT1 binds Ena/VASP proteins and is necessary for actin dynamics and cell polarization. EMBO J.

[16] Tanoue T, Takeichi M (2004). Mammalian Fat1 cadherin regulates actin dynamics and cell-cell contact. J Cell Biol.

[17] Bear JE, Svitkina TM, Krause M, Schafer DA, Loureiro JJ, Strasser GA (2002). Antagonism between Ena/VASP proteins and actin filament capping regulates fibroblast motility. Cell.

[18] Skouloudaki K, Puetz M, Simons M, Courbard JR, Boehlke C, Hartleben B (2009). Scribble participates in Hippo signaling and is required for normal zebrafish pronephros development. Proc Natl Acad Sci U S A.

[19] Morris LG, Kaufman AM, Gong Y, Ramaswami D, Walsh LA, Turcan Ş (2013). Recurrent somatic mutation of FAT1 in multiple human cancers leads to aberrant Wnt activation. Nat Genet.

[20] Campbell JD, Yau C, Bowlby R, Liu Y, Brennan K, Fan H (2018). Genomic, Pathway Network, and Immunologic Features Distinguishing Squamous Carcinomas. Cell Rep.

[21] Stransky N, Egloff AM, Tward AD, Kostic AD, Cibulskis K, Sivachenko A (2011). The mutational landscape of head and neck squamous cell carcinoma. Science.

[22] Martin D, Degese MS, Vitale-Cross L, Iglesias-Bartolome R, Valera JLC, Wang Z (2018). Assembly and activation of the Hippo signalome by FAT1 tumor suppressor. Nat Commun.

[23] Pastushenko I, Mauri F, Song Y, de Cock F, Meeusen B, Swedlund B (2021). Fat1 deletion promotes hybrid EMT state, tumour stemness and metastasis. Nature.

[24] Li Z, Razavi P, Li Q, Toy W, Liu B, Ping C (2018). Loss of the FAT1 Tumor Suppressor Promotes Resistance to CDK4/6 Inhibitors via the Hippo Pathway. Cancer Cell.

[25] Bu J, Zhang Y, Wu S, Li H, Sun L, Liu Y (2023). KK-LC-1 as a therapeutic target to eliminate ALDH(+) stem cells in triple negative breast cancer. Nat Commun.

[26] Kwaepila N, Burns G, Leong AS (2006). Immunohistological localisation of human FAT1 (hFAT) protein in 326 breast cancers. Does this adhesion molecule have a role in pathogenesis?. Pathology.

[27] Hynes RO, Naba A (2012). Overview of the matrisome-an inventory of extracellular matrix constituents and functions. Cold Spring Harb Perspect Biol.

[28] Naba A, Clauser KR, Hoersch S, Liu H, Carr SA, Hynes RO (2012). The matrisome: in silico definition and in vivo characterization by proteomics of normal and tumor extracellular matrices. Mol Cell Proteomics.

[29] Zheng SM, Feng YC, Zhu Q, Li RQ, Yan QQ, Teng L (2024). MILIP Binding to tRNAs Promotes Protein Synthesis to Drive Triple-Negative Breast Cancer. Cancer Res.

[30] Feng YC, Liu XY, Teng L, Ji Q, Wu Y, Li JM (2020). c-Myc inactivation of p53 through the pan-cancer lncRNA MILIP drives cancer pathogenesis. Nat Commun.

[31] Pontén F, Jirström K, Uhlen M (2008). The Human Protein Atlas-a tool for pathology. J Pathol.

[32] Nguyen QH, Pervolarakis N, Blake K, Ma D, Davis RT, James N (2018). Profiling human breast epithelial cells using single cell RNA sequencing identifies cell diversity. Nat Commun.

[33] Mahendralingam MJ, Kim H, McCloskey CW, Aliar K, Casey AE, Tharmapalan P (2021). Mammary epithelial cells have lineage-rooted metabolic identities. Nat Metab.

[34] Dai X, Li T, Bai Z, Yang Y, Liu X, Zhan J (2015). Breast cancer intrinsic subtype classification, clinical use and future trends. Am J Cancer Res.

[35] Hu Z, Fan C, Oh DS, Marron JS, He X, Qaqish BF (2006). The molecular portraits of breast tumors are conserved across microarray platforms. BMC Genomics.

[36] Wirapati P, Sotiriou C, Kunkel S, Farmer P, Pradervand S, Haibe-Kains B (2008). Meta-analysis of gene expression profiles in breast cancer: toward a unified understanding of breast cancer subtyping and prognosis signatures. Breast Cancer Res.

[37] Desmedt C, Haibe-Kains B, Wirapati P, Buyse M, Larsimont D, Bontempi G (2008). Biological processes associated with breast cancer clinical outcome depend on the molecular subtypes. Clin Cancer Res.

[38] Tang W, Zhou M, Dorsey TH, Prieto DA, Wang XW, Ruppin E (2018). Integrated proteotranscriptomics of breast cancer reveals globally increased protein-mRNA concordance associated with subtypes and survival. Genome Med.

[39] Burstein MD, Tsimelzon A, Poage GM, Covington KR, Contreras A, Fuqua SA (2015). Comprehensive genomic analysis identifies novel subtypes and targets of triple-negative breast cancer. Clin Cancer Res.

[40] Jézéquel P, Campone M, Gouraud W, Guérin-Charbonnel C, Leux C, Ricolleau G (2012). bc-GenExMiner: an easy-to-use online platform for gene prognostic analyses in breast cancer. Breast Cancer Res Treat.

[41] Jézéquel P, Gouraud W, Ben Azzouz F, Guérin-Charbonnel C, Juin PP, Lasla H (2021). bc-GenExMiner 4.5: new mining module computes breast cancer differential gene expression analyses. Database (Oxford).

[42] Jézéquel P, Lasla H, Gouraud W, Basseville A, Michel B, Frenel JS (2024). Mesenchymal-like immune-altered is the fourth robust triple-negative breast cancer molecular subtype. Breast Cancer.

[43] Goldman MJ, Craft B, Hastie M, Repečka K, McDade F, Kamath A (2020). Visualizing and interpreting cancer genomics data via the Xena platform. Nat Biotechnol.

[44] Hess KR, Anderson K, Symmans WF, Valero V, Ibrahim N, Mejia JA (2006). Pharmacogenomic predictor of sensitivity to preoperative chemotherapy with paclitaxel and fluorouracil, doxorubicin, and cyclophosphamide in breast cancer. J Clin Oncol.

[45] Ru B, Wong CN, Tong Y, Zhong JY, Zhong SSW, Wu WC (2019). TISIDB: an integrated repository portal for tumor-immune system interactions. Bioinformatics.

[46] Zhou C, Gao Y, Ding P, Wu T, Ji G (2023). The role of CXCL family members in different diseases. Cell Death Discov.

[47] DeNardo DG, Coussens LM (2007). Inflammation and breast cancer. Balancing immune response: crosstalk between adaptive and innate immune cells during breast cancer progression. Breast Cancer Res.

[48] Neve RM, Chin K, Fridlyand J, Yeh J, Baehner FL, Fevr T (2006). A collection of breast cancer cell lines for the study of functionally distinct cancer subtypes. Cancer Cell.

[49] Ringnér M, Fredlund E, Häkkinen J, Borg Å, Staaf J (2011). GOBO: gene expression-based outcome for breast cancer online. PLoS One.

[50] Wang R, Lv Q, Meng W, Tan Q, Zhang S, Mo X (2014). Comparison of mammosphere formation from breast cancer cell lines and primary breast tumors. J Thorac Dis.

[51] Chin YR, Yoshida T, Marusyk A, Beck AH, Polyak K, Toker A (2014). Targeting Akt3 signaling in triple-negative breast cancer. Cancer Res.

[52] Staley BK, Irvine KD (2012). Hippo signaling in Drosophila: recent advances and insights. Dev Dyn.

[53] Katoh M (2012). Function and cancer genomics of FAT family genes. Int J Oncol.

[54] Qi C, Zhu YT, Hu L, Zhu YJ (2009). Identification of Fat4 as a candidate tumor suppressor gene in breast cancers. Int J Cancer.

[55] Mao W, Zhou J, Hu J, Zhao K, Fu Z, Wang J (2022). A pan-cancer analysis of FAT atypical cadherin 4 (FAT4) in human tumors. Front Public Health.

[56] Hou L, Chen M, Zhao X, Li J, Deng S, Hu J (2016). FAT4 functions as a tumor suppressor in triple-negative breast cancer. Tumour Biol.

[57] Wojtalewicz N, Sadeqzadeh E, Weiß JV, Tehrani MM, Klein-Scory S, Hahn S (2014). A soluble form of the giant cadherin Fat1 is released from pancreatic cancer cells by ADAM10 mediated ectodomain shedding. PLoS One.

[58] Li Y, Zhang H, Merkher Y, Chen L, Liu N, Leonov S (2022). Recent advances in therapeutic strategies for triple-negative breast cancer. J Hematol Oncol.

[59] Irshad K, Srivastava C, Malik N, Arora M, Gupta Y, Goswami S (2022). Upregulation of Atypical Cadherin FAT1 Promotes an Immunosuppressive Tumor Microenvironment via TGF-β. Front Immunol.

[60] Zhang Q, Li MK, Hu XY, Wu YX, Wang YY, Zhao PP (2024). The tumour suppressor Fat1 is dispensable for normal murine hematopoiesis. J Leukoc Biol.

[61] Chen C, Li Y, Liu H, Liao M, Yang J, Liu J (2024). FAT1 upregulation is correlated with an immunosuppressive tumor microenvironment and predicts unfavorable outcome of immune checkpoint therapy in non-small cell lung cancer. Heliyon.

[62] Zhu W, Yang L, Gao Y, Zhou Y, Shi Y, Liu K (2024). Clinical value of FAT1 mutations to indicate the immune response in colorectal cancer patients. Genomics.

[63] Zhu K, Wu Y, He P, Fan Y, Zhong X, Zheng H (2022). PI3K/AKT/mTOR-Targeted Therapy for Breast Cancer. Cells.

[64] Grasset EM, Dunworth M, Sharma G, Loth M, Tandurella J, Cimino-Mathews A (2022). Triple-negative breast cancer metastasis involves complex epithelial-mesenchymal transition dynamics and requires vimentin. Sci Transl Med.

[65] Hinz N, Jücker M (2019). Distinct functions of AKT isoforms in breast cancer: a comprehensive review. Cell Commun Signal.

[66] Li S, Sampson C, Liu C, Piao HL, Liu HX (2023). Integrin signaling in cancer: bidirectional mechanisms and therapeutic opportunities. Cell Commun Signal.

[67] Hynes RO (2002). Integrins: bidirectional, allosteric signaling machines. Cell.

[68] Luo J, Yao JF, Deng XF, Zheng XD, Jia M, Wang YQ (2018). 14, 15-EET induces breast cancer cell EMT and cisplatin resistance by up-regulating integrin αvβ3 and activating FAK/PI3K/AKT signaling. J Exp Clin Cancer Res.

[69] Madan E, Dikshit B, Gowda SH, Srivastava C, Sarkar C, Chattopadhyay P (2016). FAT1 is a novel upstream regulator of HIF1α and invasion of high grade glioma. Int J Cancer.

[70] Madamanchi A, Zijlstra A, Zutter MM (2014). Flipping the switch: integrin switching provides metastatic competence. Sci Signal.

[71] Truong HH, Xiong J, Ghotra VP, Nirmala E, Haazen L, Le Dévédec SE (2014). β1 integrin inhibition elicits a prometastatic switch through the TGFβ-miR-200-ZEB network in E-cadherin-positive triple-negative breast cancer. Sci Signal.

[72] Hwang PY, Mathur J, Cao Y, Almeida J, Ye J, Morikis V (2023). A Cdh3-β-catenin-laminin signaling axis in a subset of breast tumor leader cells control leader cell polarization and directional collective migration. Dev Cell.

[73] Sun Q, Wang Y, Officer A, Pecknold B, Lee G, Harismendy O (2022). Stem-like breast cancer cells in the activated state resist genetic stress via TGFBI-ZEB1. NPJ Breast Cancer.

[74] Cao LL, Riascos-Bernal DF, Chinnasamy P, Dunaway CM, Hou R, Pujato MA (2016). Control of mitochondrial function and cell growth by the atypical cadherin Fat1. Nature.

[75] de Bock CE, Thorne RF (2016). Cell biology: A mitochondrial brake on vascular repair. Nature.

[76] Chandrashekar DS, Karthikeyan SK, Korla PK, Patel H, Shovon AR, Athar M (2022). UALCAN: An update to the integrated cancer data analysis platform. Neoplasia.

[77] Cerami E, Gao J, Dogrusoz U, Gross BE, Sumer SO, Aksoy BA (2012). The cBio cancer genomics portal: an open platform for exploring multidimensional cancer genomics data. Cancer Discov.

[78] Chung W, Eum HH, Lee HO, Lee KM, Lee HB, Kim KT (2017). Single-cell RNA-seq enables comprehensive tumour and immune cell profiling in primary breast cancer. Nat Commun.

