# Supplementary material for: FAT1 functions as an oncogenic driver in triple negative breast cancer through AKT pathway-driven effects on the matrisome
Source: Int J Biol Sci. 2025 Feb 24;21(5):2201–22. doi: 10.7150/ijbs.104921 (PMC11900804; doi:10.7150/ijbs.104921)
Supplement: Supplementary file 1 — Supplementary figures. [file ijbsv21p2201s1.pdf]

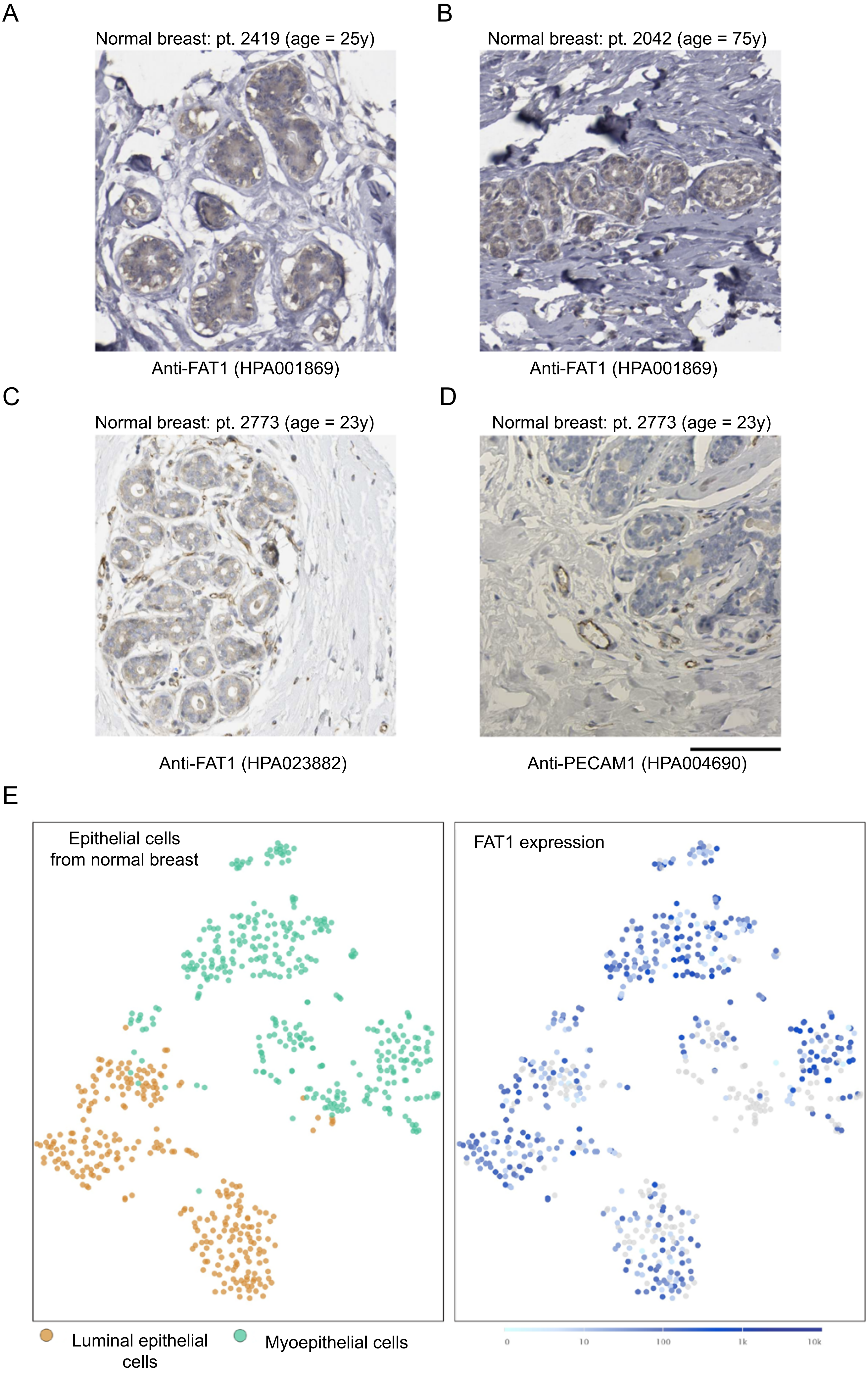

Supplementary Figure 1

F

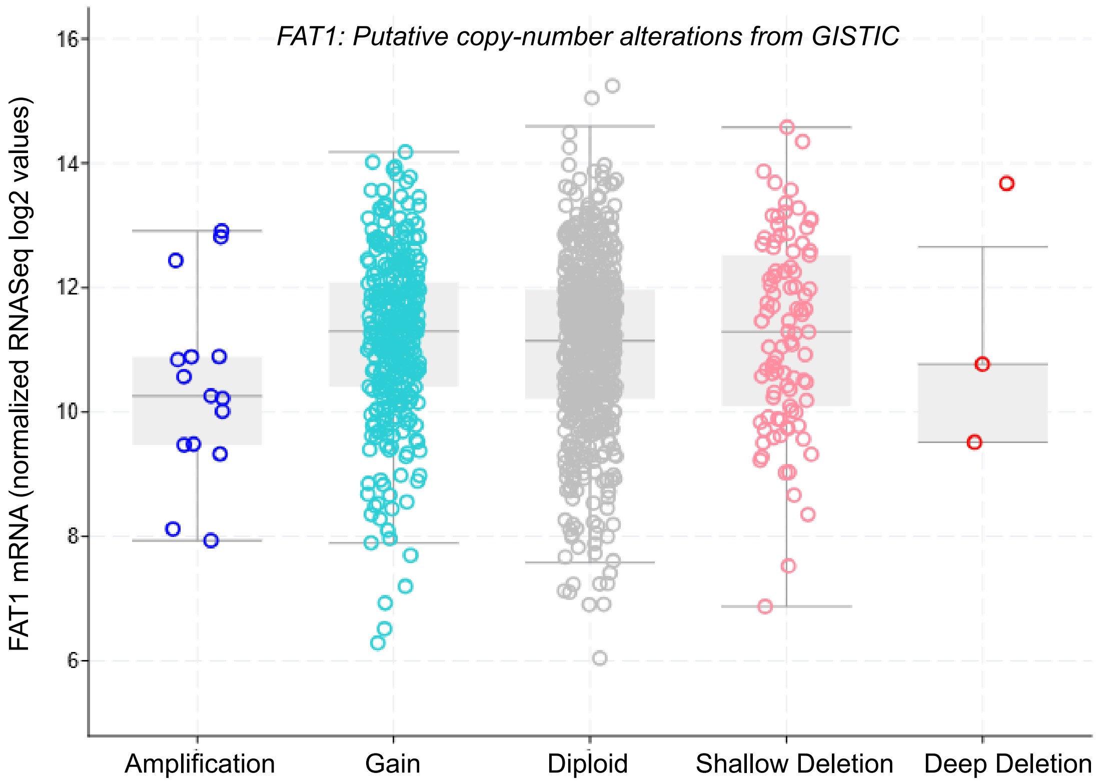

Figure S1: FAT1 expression in normal breast cells and tissues (related to Fig. 1).

(A-D) Immunohistochemical staining of normal breast tissues with antibodies directed against the C-terminal domain of FAT1 (A, B), the N-terminal domain of FAT1 (C) or antibodies decorating the endothelial marker PECAM-1 (D) (bar = 200 um; images derived from the Human Protein Atlas (proteinatlas.org).

(E) Single cell analysis of FAT1 expression in normal breast epithelial cells. tSNE plot showing cell type clusters defining luminal and basal epithelial cells isolated from adult human breast epithelium (631 cells from n=3 patients; left) with a corresponding plot of FAT1 expression (ENSG00000083857; right). Data derived from the Nguyen study interrogated using the EM-BL-EBI Single Cell Expression Atlas (ebi.ac.uk).

(F) Relationship between FAT1 mRNA expression levels and FAT1 gene copy number alterations in primary breast cancer tissue samples from the TCGA Breast Invasive Carcinoma dataset plotted using cBioportal (www.cbioportal.org).

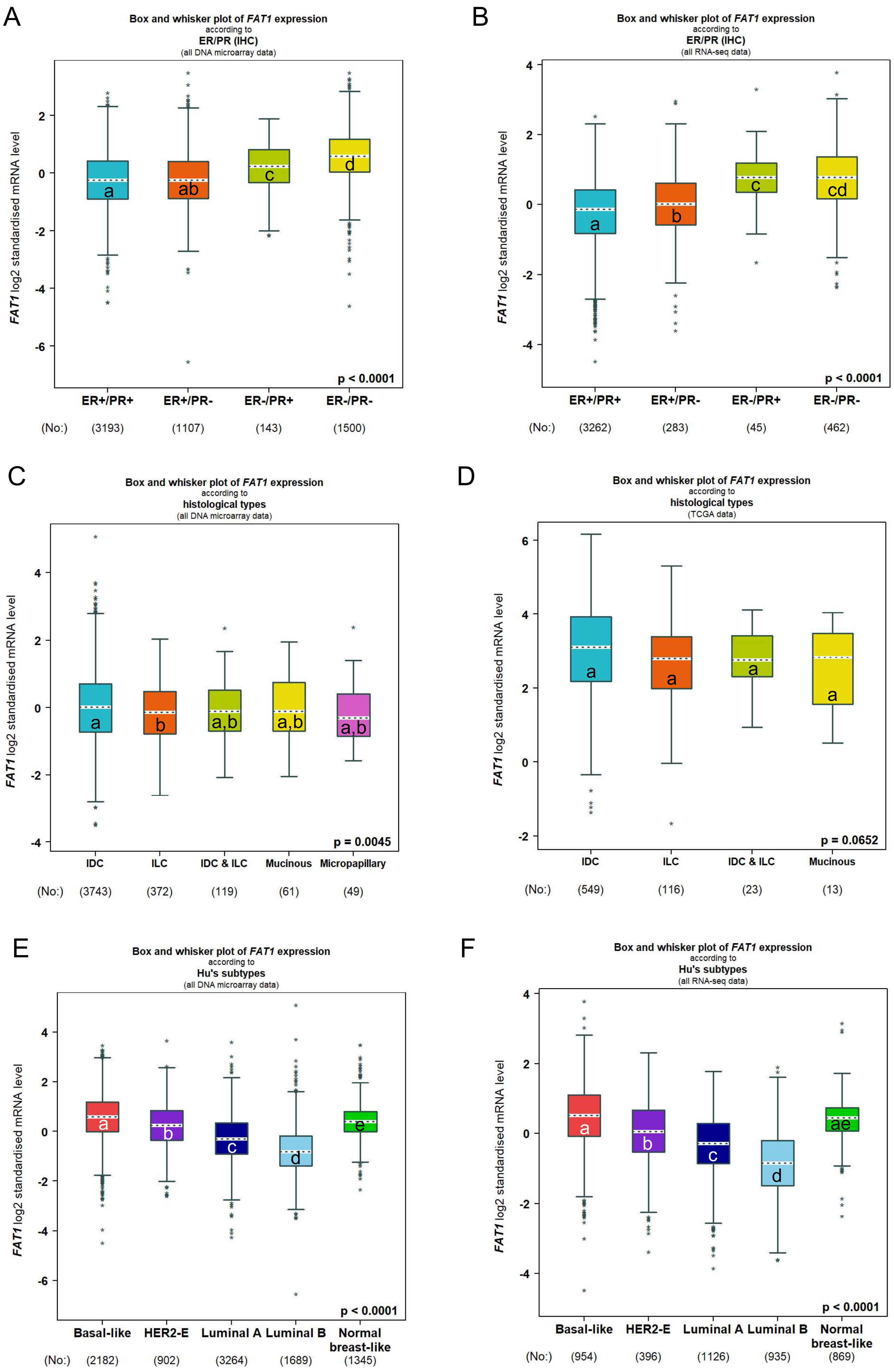

Supplementary Figure 2

G H

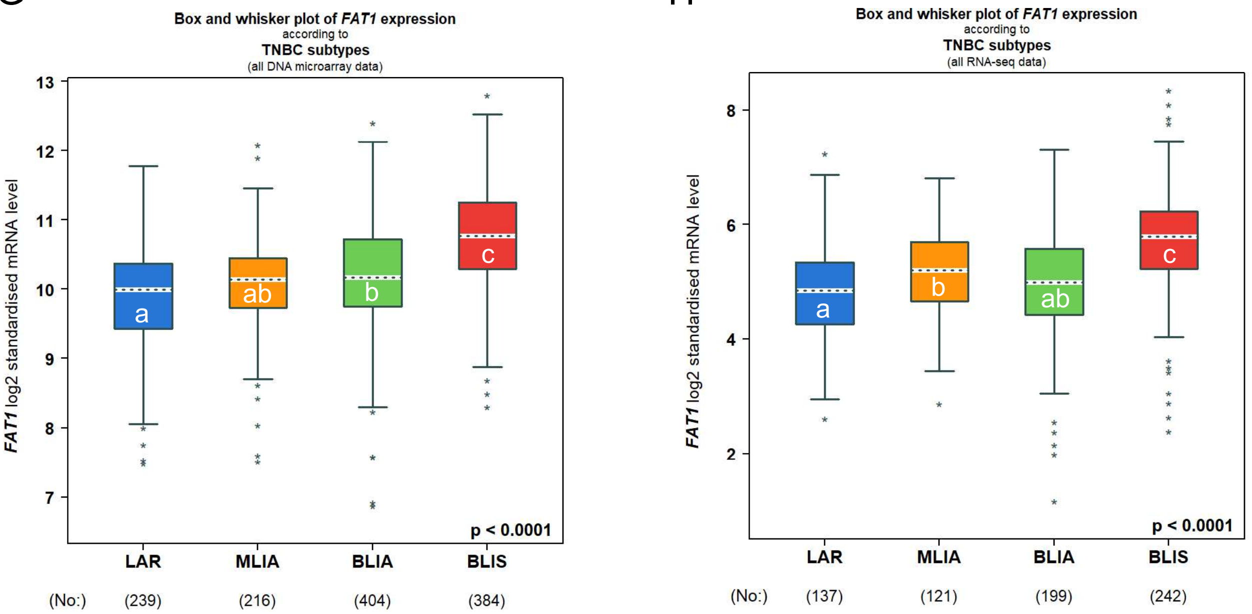

Figure S2: Comparative FAT1 expression among pathological, histological and molecular classifications of breast cancer (related to Figure 2).

(A, B) FAT1 mRNA expression in breast cancer classified by ER/PR expression status from DNA microarray (A) and RNA-seq (B) based studies. Plots prepared using the Breast Cancer Gene-Expression Miner v5.0 (bc-GenExMiner v5.0: http://bcgenex.ico.unicancer.fr/).

(C, D) FAT1 mRNA expression in breast cancer classified by histological subtypes from DNA microarray (C) and RNA-seq (D) based studies. Plots prepared using bc-GenExMiner v5.0. Invasive ductal carcinoma (IDC); invasive lobular carcinoma (ILC).

(E, F) FAT1 mRNA expression in breast cancer classified by molecular subtypes according to Hu's definitions from DNA microarray (E) and RNA-seq (F) based studies. Plots prepared using bc-GenExMiner v5.0.

(G, H) FAT1 mRNA expression among molecular subtypes of TNBC determined from DNA microarray (G) and RNA-seq (H) based studies. Plots prepared using bc-GenExMiner v5.0.

(A-G) Statistical differences determined using one-way ANOVA with post-hoc Dunnett-Tukey-Kramer tests used to define inter-group comparisons. Means are not statistically significant for columns marked with the same letter.

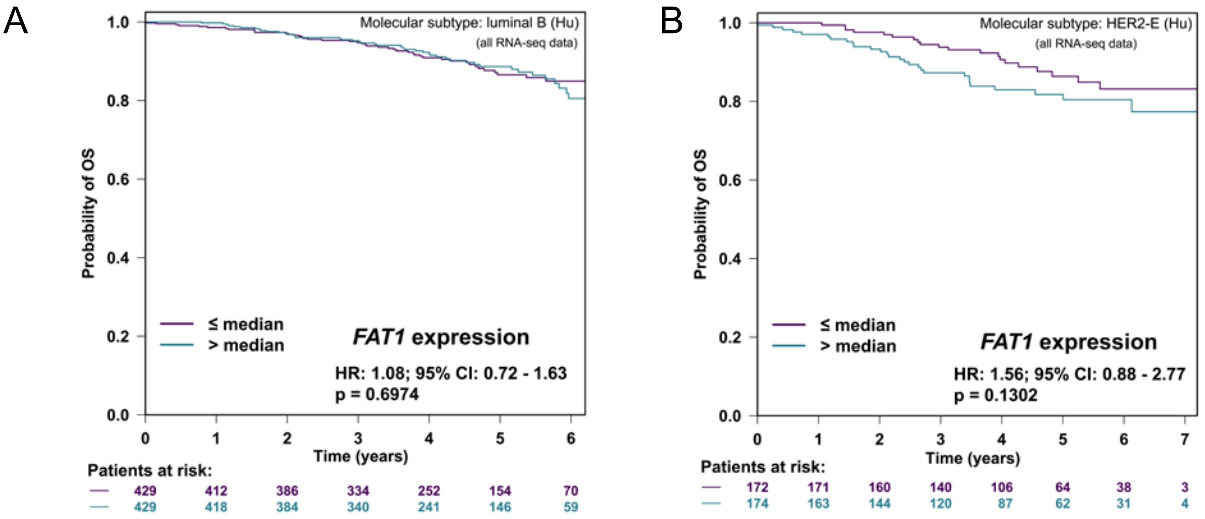

C

***FAT1* univariate Cox analysis by SSP molecular subtype**
(all DNA microarray data - OS - median split)

Subtype	Gene expression signature	p-value	HR	95% CI	Good prognosis' RNA level	No. patients	No. events
Luminal A	1 Hu	< 0.0001	0.73	0.63 - 0.83	↗	1 932	858
	2 Sorlie	< 0.0001	0.68	0.57 - 0.82	↗	1 173	485
	3 PAM50	< 0.0001	0.77	0.68 - 0.87	↗	2 346	1 071
	4 RSSPC	0.0054	0.72	0.57 - 0.91	↗	763	328
Basal-like	1 Hu	< 0.0001	1.57	1.26 - 1.95	↘	901	386
	2 PAM50	0.0121	1.32	1.06 - 1.64	↘	883	369
	3 Sorlie	0.1190	1.22	0.95 - 1.56	--	613	278
	4 RSSPC	0.1413	1.22	0.94 - 1.58	--	542	243
Luminal B	1 RSSPC	0.0621	1.56	0.98 - 2.48	↘	152	84
	2 Hu	0.4163	1.10	0.87 - 1.39	--	665	318
	3 PAM50	0.6357	1.05	0.85 - 1.32	--	815	365
	4 Sorlie	0.9205	1.01	0.87 - 1.16	--	1 424	783
HER2-E	1 PAM50	0.1248	1.21	0.95 - 1.54	--	652	296
	2 Sorlie	0.3070	1.21	0.84 - 1.74	--	354	142
	3 Hu	0.3875	1.14	0.84 - 1.55	--	428	210
	4 RSSPC	0.9719	0.99	0.55 - 1.79	--	125	56

D

***FAT1* univariate Cox analysis by SSP molecular subtype:**
(all RNA-seq data - OS - median split)

Subtype	Gene expression signature	p-value	HR	95% CI	Good prognosis' RNA level	No. patients	No. events
Luminal A	1 PAM50	0.0057	0.55	0.36 - 0.84	↗	1 205	92
	2 RSSPC	0.0153	0.49	0.27 - 0.87	↗	632	51
	3 Hu	0.0431	0.65	0.42 - 0.99	↗	1 022	88
	4 Sorlie	0.2511	0.81	0.57 - 1.16	--	1 359	127
Basal-like	1 Hu	0.0082	1.58	1.13 - 2.22	↘	865	138
	2 PAM50	0.0153	1.59	1.09 - 2.33	↘	712	112
	3 Sorlie	0.0971	1.42	0.94 - 2.16	↘	521	90
	4 RSSPC	0.4621	1.17	0.77 - 1.78	--	488	88
Luminal B	1 PAM50	0.3977	1.19	0.80 - 1.77	--	891	98
	2 Hu	0.6974	1.08	0.72 - 1.63	--	858	94
	3 Sorlie	0.8307	1.05	0.65 - 1.72	--	571	66
	4 RSSPC	0.8972	0.94	0.39 - 2.30	--	194	22
HER2-E	1 PAM50	0.0852	1.45	0.95 - 2.21	↘	619	92
	2 Hu	0.1302	1.56	0.88 - 2.77	--	346	49
	3 RSSPC	0.4398	1.35	0.63 - 2.90	--	172	27
	4 Sorlie	0.7275	0.93	0.60 - 1.42	--	548	85

Figure S3: FAT1 expression and breast cancer patient prognosis (related to Figure 3).
(A, B) Kaplan-Meier plots of OS in patients stratified according to median FAT1 mRNA expression in the Luminal B (A) and HER2 molecular subtypes (B) classified using Hu's definitions. Plots prepared with bc-GenExMiner v5.0 from RNA-seq based studies.
(C, D) Summary tables of Cox univariate analyses of OS among breast cancer molecular subtypes classified by median FAT1 expression in DNA microarray (C) and RNA-seq (D) based studies. Comparative statistical data obtained using molecular subtypes according to the Hu, Sorlie, PAM50 (Parker's molecular 50) and RSSPC (robust single sample predictor classification) definitions.

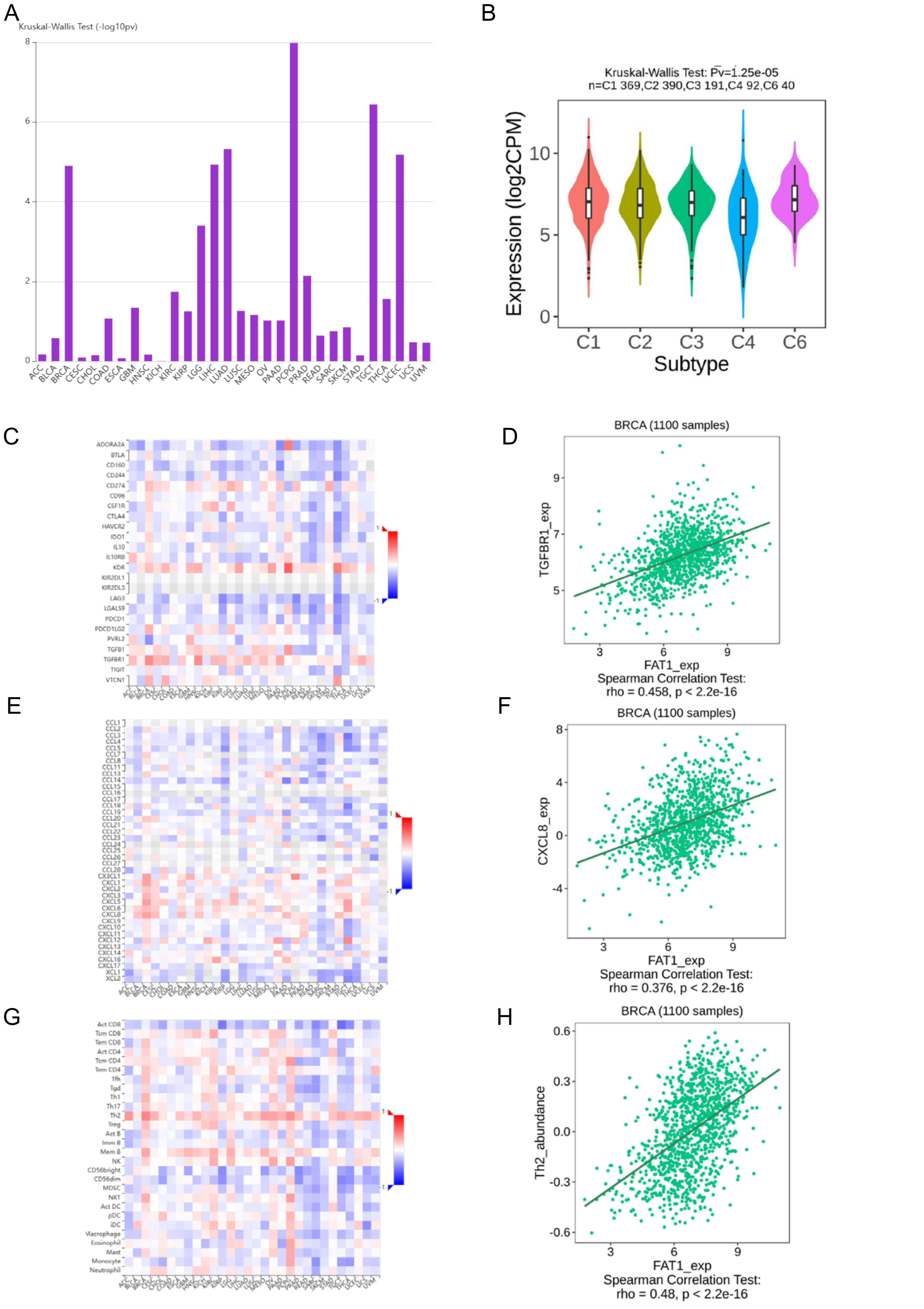

Supplementary Figure 4

Figure S4: Associations between FAT1 expression and tumor-immune system interactions (related to Figure 4).

(A, B) Relative association scores between FAT1 mRNA expression and immune cell signatures within TCGA cancer type datasets (A). Violin plots comparing FAT1 expression values in breast cancer cases with defined tumor immune subtype classifications (C1-C4, C6) (B).

(C-H) Heatmaps of Spearman correlation values (C, E, G) illustrating associations between FAT1 expression and that of defined immunomodulator genes (C), chemokines (E), and gene signatures for tumor infiltrating immune subsets (G) among TCGA cancer types. Selected plots for breast cancer illustrate correlations between FAT1 and TGF-βR1 expression (D), FAT1 and CXCL8 expression (F), and FAT1 expression and Th2 cells (F), respectively, among TCGA BRCA cases. All plots were derived using TISIDB (cis.hku.hk/TISIDB/).

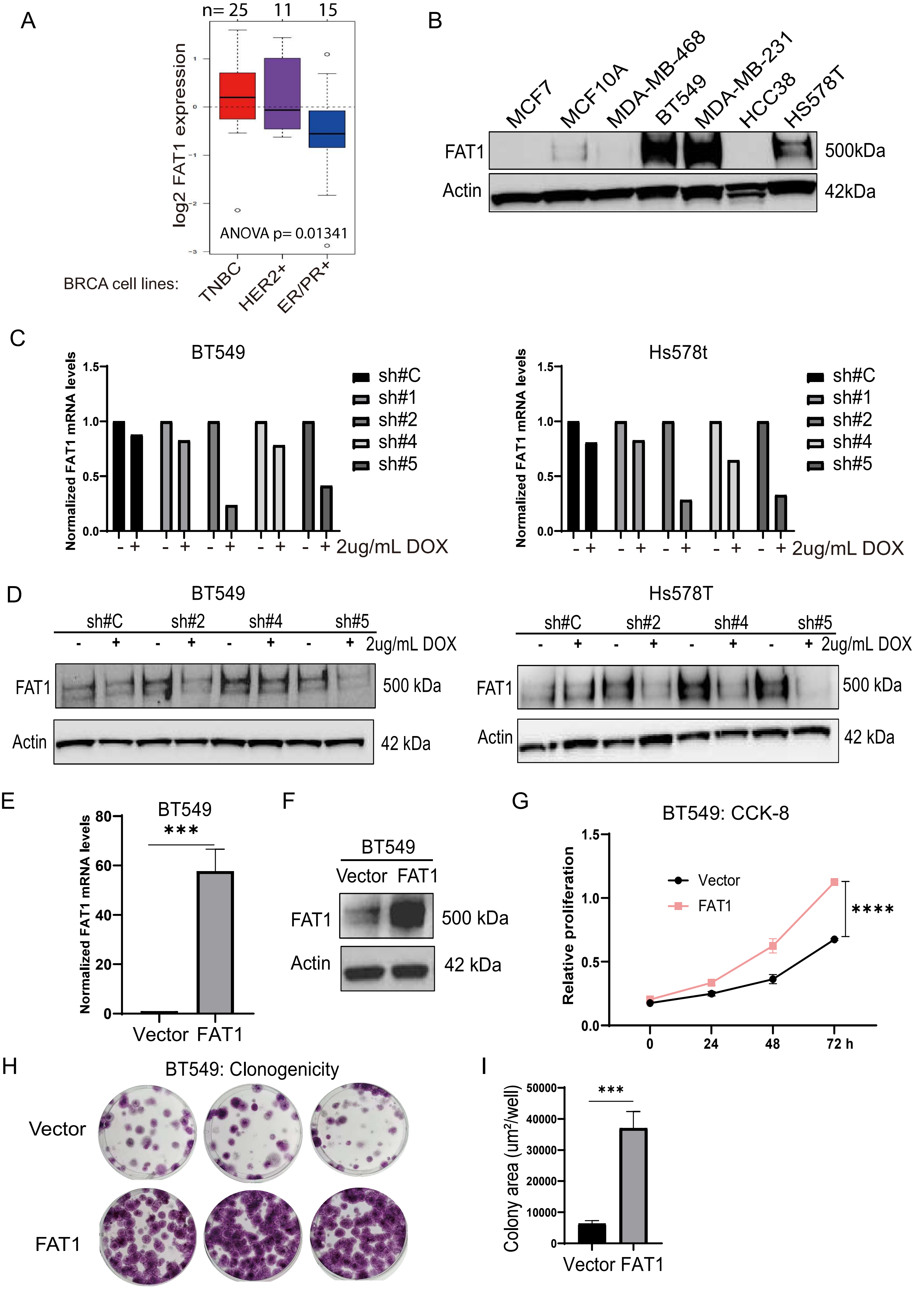

Supplementary Figure 5

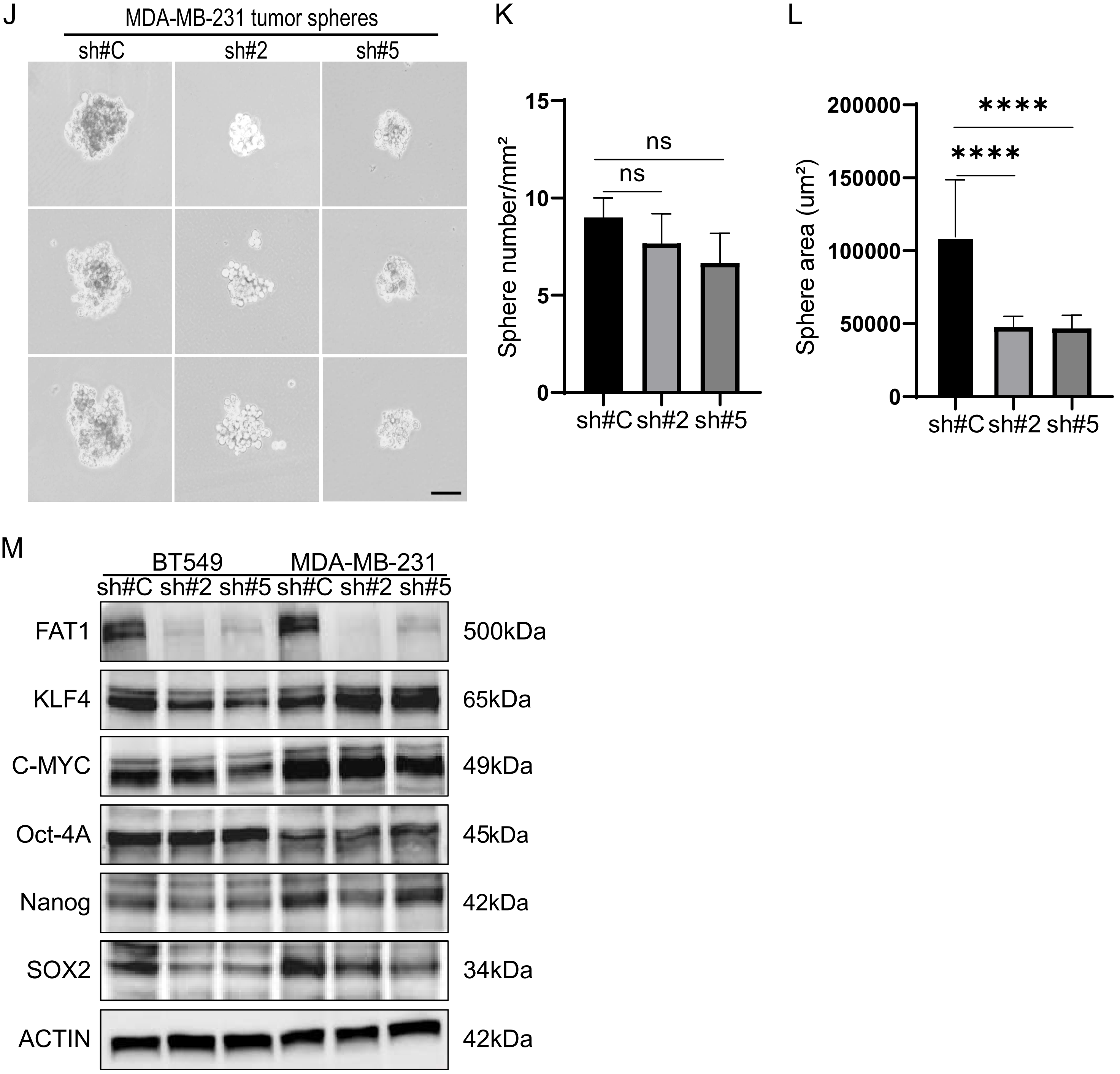

Figure S5: FAT1 knockdown and overexpression in TNBC cell lines (related to Figure 5).
(A) Box and Whisker plot comparing FAT1 mRNA expression among 51 human breast cancer cell lines classified into TNBC, HER2+ and ER/PR+ subtypes. Derived from the GOBO database (Gene expression-based Outcome for Breast cancer Online version 1.0.3; co.bmc.lu.se/gobo/gobo.pl) using data from Neve et al. (2006).
(B) Western blot against FAT1 and actin loading control using MCF10A mammary and breast cancer cell lines.
(C, D) FAT1 mRNA (C) and protein (D) levels measured by qPCR and Western blot, respectively, in BT549 (left) and Hs578t (right) cell lines transduced with doxycycline (DOX) inducible shRNAs targeting FAT1 (sh#1, sh#2, sh#4, sh#5) or a control shRNA (sh#C).
(E, F) The levels of FAT1 mRNA (E) and protein (F) were measured by qPCR and Western blot, respectively, in BT549 cells transfected with a full-length FAT1 overexpression construct. The qPCR primers used specifically recognize transfected FAT1 and not endogenous FAT1.
(G-I) The proliferative capacity of cells from (E, F) measured in CCK-8 assays (G) and colony formation assays. Representative colony images (H) and quantitation of colony growth (I).
(J-L) Tumor sphere assays conducted in MDA-MB-231 cells transduced with an shRNA control or after FAT1-knockdown in the presence of DOX. Representative images of tumor spheres (J) and quantitation of the number (K) and size of spheres (L).
Reproducibility and statistical tests. (B-L) Results representative of three independent experiments. (E, G, I, K, L) Data shown as mean ± SD of three replicates with statistical differences analyzed by Student's t test (E, I, K, L),

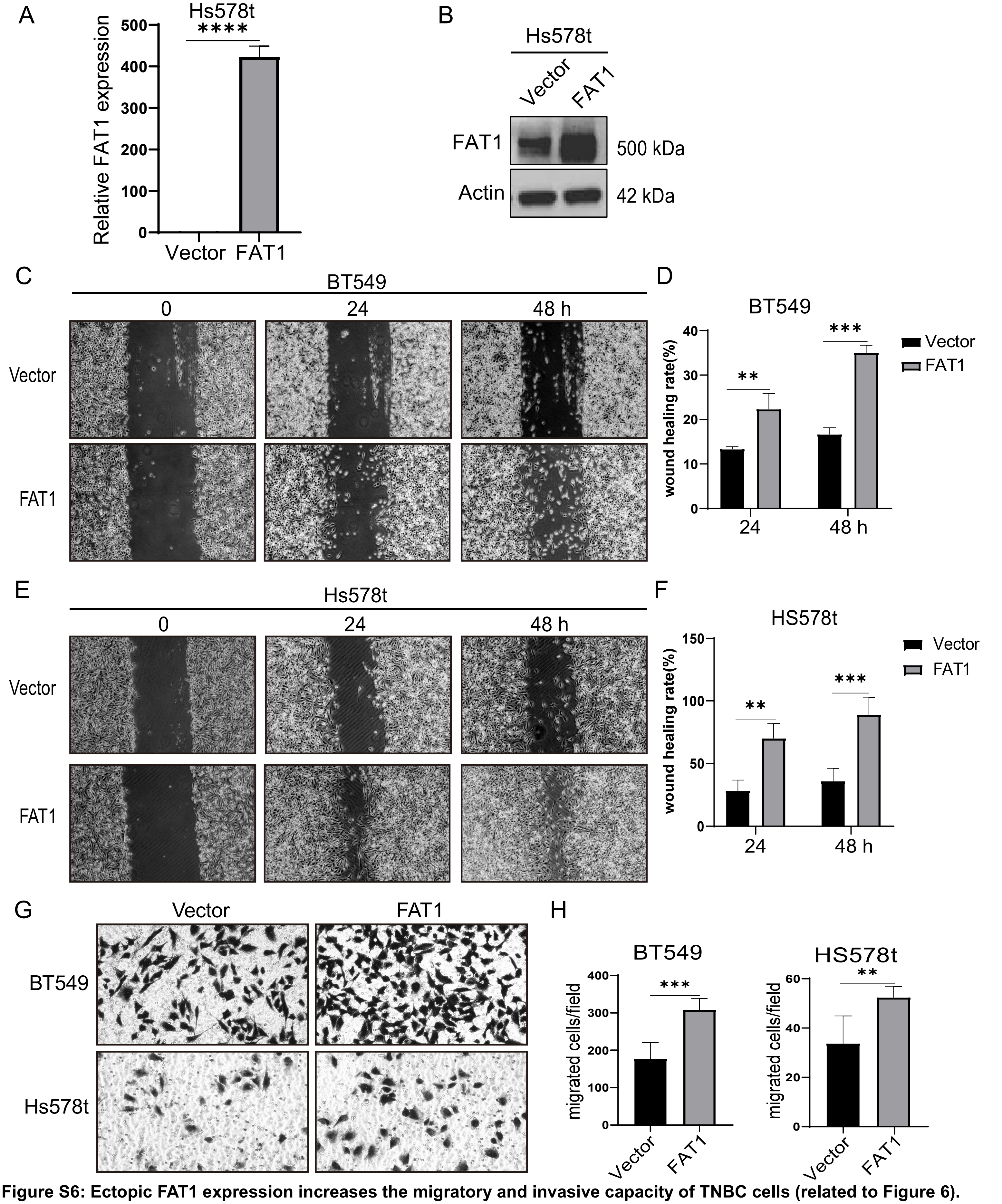

Figure S6: Ectopic FAT1 expression increases the migratory and invasive capacity of TNBC cells (related to Figure 6).
(A, B) The levels of FAT1 mRNA (A) and protein (B) were measured by qPCR and Western blot, respectively, in Hs578t cells transfected with a full-length FAT1 overexpression construct or a control vector. The qPCR primers used specifically recognize transfected FAT1 and not endogenous FAT1.
(C-F) Wound healing assays measuring cell motility were conducted on the BT549 (C, D) and Hs578t (E, F) cell lines overexpressing FAT1 or control cells. Representative images collected of the same area at 0, 24 and 48h (C, E) and quantitation of cell migration distance as wound healing rate (D, F).
(G, H) Transwell migration assays measuring cell invasion were conducted on the BT549 and Hs578t cell lines from (C-F). Migrating cells were fixed and stained after 24h and images collected (G) and quantitated as the number of cells per microscopic field (H).
Reproducibility and statistical tests. (A-H) Results representative of three independent experiments. (D, F, H) Data shown as mean ± SD of three replicates with statistical differences analyzed by Student's t test (H). $^{**}P < 0.01$; $^{***}P < 0.001$.

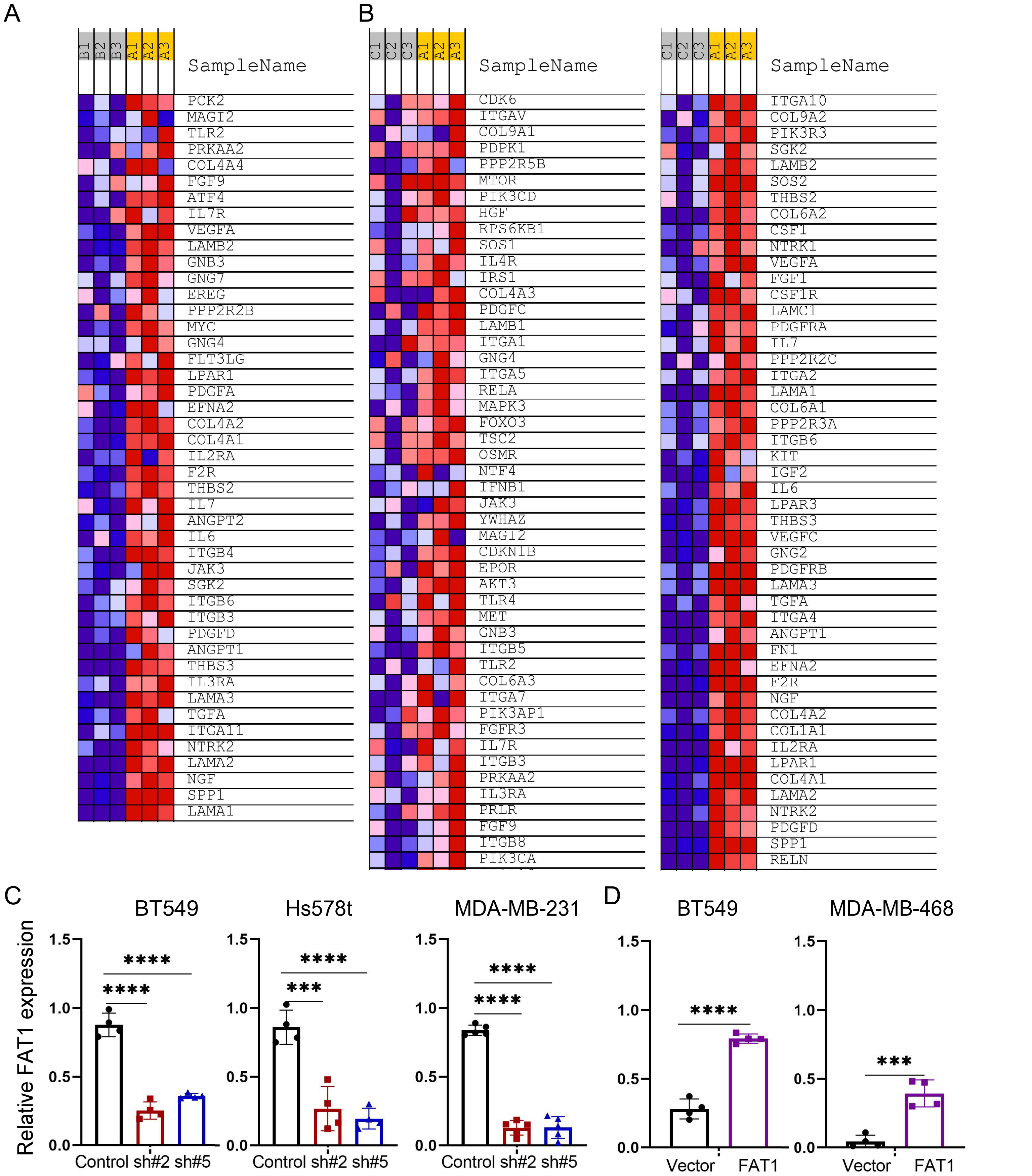

Figure S7: Gene level changes in the PI3K-AKT pathway signature following FAT1 knockdown (related to Figure 7).

(A, B) Heat maps denoting significant gene expression changes associated with the GSEA enrichment of the PI3K_AKT-SIGNALLING_PATHWAY following knockdown of FAT1 in BT549 cells. Comparisons of shCtrl (A1, A2, A3) versus shFAT1#2 (B1, B2, B3) (A) and shCtrl (A1, A2, A3) versus shFAT1#5 (C1, C2, C3) (B).

(C, D) Relative changes in FAT1 levels determined by densitometry against actin loading controls from knockdown (C) and overexpression (D) experiments in Figure 7E, H, respectively. Data are mean ± SD of three or more experiments with statistical differences analyzed by one way ANOVA (C) or Student’s t test (D). ***P < 0.001; ****P < 0.0001.

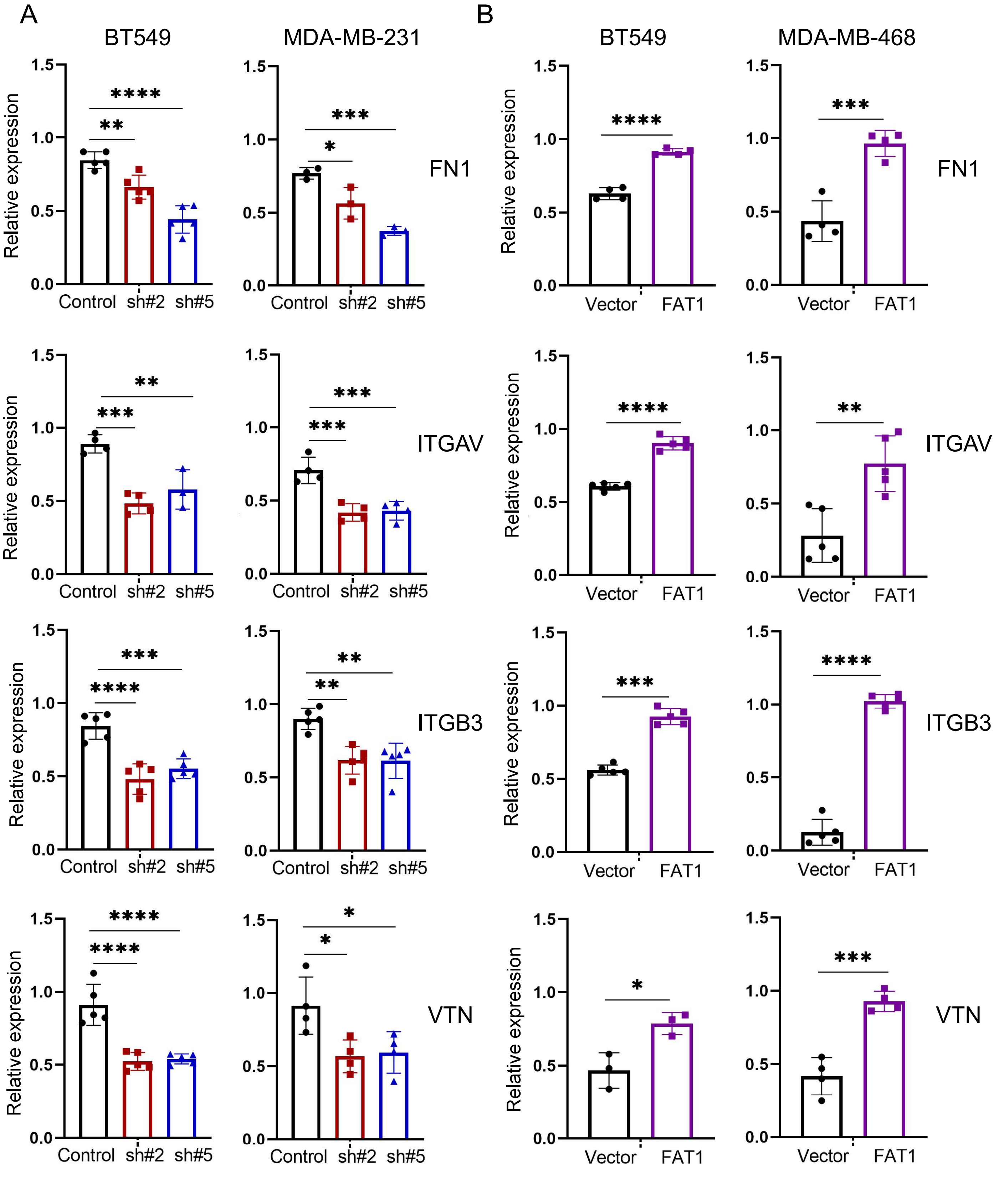

Figure S8: Changes in matrisome-related protein expression following FAT1 knockdown and overexpression in TNBC cells (related to Figure 10).

(**A, B**) Comparative changes in matrisome-related proteins (FN1, ITGAV, ITGB3, and VTN) in BT549 and MDA-MB-231 cells subject to FAT1 shRNA-mediated knockdown (A) or in BT549 and MDA-MB-468 cells after-FAT1 overexpression (B). Protein expression was determined using Western blotting with band densities normalized against the actin loading control. Data represent the mean ± SD from 3 or more experiments as indicated. Statistical differences were analyzed by one way ANOVA with posthoc Dunnett's multiple comparisons test (A) or Student's t test (B). *P < 0.05; **P < 0.01; ***P < 0.001; ****P < 0.0001.
